# Reduced peptidoglycan synthesis capacity impairs growth of *E. coli* at high salt concentration

**DOI:** 10.1128/mbio.00325-24

**Published:** 2024-03-01

**Authors:** Dema Alodaini, Victor Hernandez-Rocamora, Gabriela Boelter, Xuyu Ma, Micheal B. Alao, Hannah M. Doherty, Jack A. Bryant, Patrick Moynihan, Danesh Moradigaravand, Monika Glinkowska, Waldemar Vollmer, Manuel Banzhaf

**Affiliations:** 1 Institute of Microbiology and Infection, School of Biosciences, University of Birmingham, Birmingham, United Kingdom; 2 Biosciences Institute, Faculty of Medical Sciences, Newcastle University, Newcastle upon Tyne, United Kingdom; 3 School of Life Sciences, University of Nottingham, Nottingham, United Kingdom; 4 KAUST Computational Bioscience Research Center, King Abdullah University of Science and Technology, Thuwal, Makkah, Saudi Arabia; 5 Laboratory for Infectious Disease Epidemiology, KAUST Smart-Health Initiative and Biological and Environmental Science and Engineering Division, King Abdullah University of Science and Technology, Thuwal, Makkah, Saudi Arabia; 6 Department of Bacterial Molecular Genetics, Faculty of Biology, University of Gdańsk, Gdańsk, Poland; 7 Institute for Molecular Bioscience, University of Queensland, Brisbane, Australia; Fred Hutchinson Cancer Center, Seattle, USA

**Keywords:** bacterial cell envelope, peptidoglycan, penicillin-binding proteins, peptidoglycan hydrolases, salt stress

## Abstract

Gram-negative bacteria have a thin peptidoglycan layer between the cytoplasmic and outer membranes protecting the cell from osmotic challenges. Hydrolases of this structure are needed to cleave bonds to allow the newly synthesized peptidoglycan strands to be inserted by synthases. These enzymes need to be tightly regulated and their activities coordinated to prevent cell lysis. To better understand this process in *Escherichia coli*, we probed the genetic interactions of *mrcA* (encodes PBP1A) and *mrcB* (encodes PBP1B) with genes encoding peptidoglycan amidases and endopeptidases in envelope stress conditions. Our extensive genetic interaction network analysis revealed relatively few combinations of hydrolase gene deletions with reduced fitness in the absence of PBP1A or PBP1B, showing that none of the amidases or endopeptidases is strictly required for the functioning of one of the class A PBPs. This illustrates the robustness of the peptidoglycan growth mechanism. However, we discovered that the fitness of ∆*mrcB* cells is significantly reduced under high salt stress and *in vitro* activity assays suggest that this phenotype is caused by a reduced peptidoglycan synthesis activity of PBP1A at high salt concentration.

**IMPORTANCE:**

*Escherichia coli* and many other bacteria have a surprisingly high number of peptidoglycan hydrolases. These enzymes function in concert with synthases to facilitate the expansion of the peptidoglycan sacculus under a range of growth and stress conditions. The synthases PBP1A and PBP1B both contribute to peptidoglycan expansion during cell division and growth. Our genetic interaction analysis revealed that these two penicillin-binding proteins (PBPs) do not need specific amidases, endopeptidases, or lytic transglycosylases for function. We show that PBP1A and PBP1B do not work equally well when cells encounter high salt stress and demonstrate that PBP1A alone cannot provide sufficient PG synthesis activity under this condition. These results show how the two class A PBPs and peptidoglycan hydrolases govern cell envelope integrity in *E. coli* in response to environmental challenges and particularly highlight the importance of PBP1B in maintaining cell fitness under high salt conditions.

## INTRODUCTION

Gram-negative bacteria have a thin peptidoglycan (PG) layer between the cytoplasmic and outer membranes, protecting the cell against bursting due to turgor (1, 2). PG is composed of glycan chains connected by peptides, forming a mesh-like sacculus. Growing and dividing cells expand their PG layer by inserting new PG into the sacculus through the combined activities of synthases and hydrolases (3, 4). Most bacteria have multiple PG synthases. Glycosyltransferases (GTases) polymerize PG chains from the PG precursor lipid II, and this activity is provided by shape, elongation, division, and sporulation proteins (SEDS) (5), monofunctional glycosyltransferases (6), and class A penicillin-binding proteins (PBPs). dd-Transpeptidases (TPases) cross-link peptides linked to adjacent chains, and this activity is present in both class A PBPs and class B PBPs (7). Class B PBPs are monofunctional dd-transpeptidases.

In *Escherichia coli,* the class A PBPs, PBP1A and PBP1B, are responsible for synthesizing a major portion of new PG (8, 9). The depletion of both PBPs is lethal, but the absence of one of them only slightly affects cell growth or morphology, suggesting redundant roles (10, 11). In agreement with this, both PBP1A and PBP1B were identified as components of the two different elongasomes present in *Salmonella enterica* (12). Both PBPs require a different outer membrane-anchored lipoprotein activator for functioning in the cell—LpoA for PBP1A and LpoB for PBP1B (4, 13). There is evidence that both PBPs may have preferred cellular roles. PBP1A interacts with PBP2, which is needed for cell elongation (14), whereas PBP1B interacts with PBP3, which is essential for cell division (15). In addition, both PBPs have different functionality under stress conditions. In a recent study, *E. coli* MG1655Δ*mrcB* cells (*mrcB* encodes for PBP1B) showed a strong growth defect at acidic pH and cells lacking *mrcA* (which encodes for PBP1A) are sick at alkaline pH (16). In addition, *mrcB* mutants are sensitive to osmotic stress caused by sucrose (17).

PG hydrolases are also needed for sacculus growth, facilitating the insertion of the new PG strands during cell elongation and cleavage of septal PG for daughter cell separation during cell division (3). The activities of PG synthases and hydrolases need to be tightly regulated and coordinated to allow for accurate expansion of the sacculus and to prevent cell lysis (18–20). This coordination is likely achieved by protein-protein interactions of PG synthases, hydrolases, and/or regulator proteins. We do not currently know whether PBP1A or PBP1B can interchangeably work with the entire arsenal of periplasmic PG cleaving enzymes (autolysins) in *E. coli* though several protein-protein interactions have been observed (14, 15, 21, 22). This broad grouping of enzymes includes at least four *N*-acetylmuramoyl-l-alanine amidases, seven endopeptidases (EPases), and eight lytic transglycosylases (23, 24). Amidases remove peptides from the glycan strands, and in this study, we focused on the three well-described amidases crucial for septation, AmiA, AmiB, and AmiC. Amidases are autoinhibited and activated by EnvC (AmiA and AmiB) (25–29), NlpD (AmiC) (30), and ActS (AmiA, AmiB, and AmiC) (31, 32). We also included the main d,d-EPases, which hydrolyse d,d-cross-links. These are MepA, MepM, MepH, MepS, PBP4, and PBP7 (33–36).

To test whether *E. coli* PBP1A and PBP1B may require specific hydrolases to incorporate the new material into the sacculus, we probed the genetic interactions of *mrcA* and *mrcB* under various conditions leading to envelope stresses in *E. coli*. Our analysis shows that the genetic interaction network between the class A PBPs, the amidases, and endopeptidases lacks strongly interacting pairs, and hence, none of the hydrolases is specifically required for PBP1A or PBP1B function. However, we discovered that the fitness of ∆*mrcB* cells is significantly reduced under high salt stress and confirmed that this phenotype is likely caused by a reduced PG synthesis activity of PBP1A at high salt concentration. Our results reinforce the hypothesis that redundancy in periplasmic PG enzymes is required for *E. coli* to thrive under a wide range of stresses (16, 32, 37, 38).

## RESULTS

### Salt stress reduces the fitness of *E. coli* ∆*mrcB* (PBP1B) mutants

In this study, we explored whether the two major *E. coli* PG synthases PBP1A and PBP1B have preferred (genetic) interactions with or are dependent on certain PG endopeptidases and amidases, or if these PG synthases and hydrolases can function interchangeably with each other.

We first tested whether double mutants lacking a PG hydrolase and either *mrcA* (PBP1A) or *mrcB* (PBP1B) had an altered cell morphology. We generated a panel of single mutants lacking amidases/regulators (*amiA, amiB, amiC, nlpD, envC*) or EPases (*mepA, mepS, mepM, mepH, pbpG, dacB*), and double mutants in combination with ∆*mrcA* and ∆*mrcB*, by P1 transduction into the *E. coli* BW25113 background (WT, wildtype) (Table S1). We were unable to generate a ∆*envC*∆*mrcB* mutant, suggesting that the simultaneous deletion of *envC* and *mrcB* may be synthetically lethal, and we are currently exploring this observation in a separate study.

We next analyzed the morphology of all mutant cells using phase contrast and fluorescence microscopy. We grew the mutants to mid-exponential phase under standard laboratory conditions for *E. coli* (37°C, LB medium, pH 6.9). Cells were stained with both membrane dye (FM-143 FX) and DNA dye (DAPI), and the images were analyzed as described in the Materials and Methods to quantify septa, cell length, and cell width.

All single mutants showed wildtype-like or mild abnormal morphologies in accordance with previously published work (21, 39) and with our data for the WT strain (average length 3.6 ± 0.9 µm and average width 0.99 ± 0.1 µm) (Fig. S1). However, some of the double mutants showed mild changes in morphology compared to their parental mutants (Fig. S1 and S2; Table S2). Cells of ∆*mepS*∆*mrcA* and ∆*mepS*∆*mrcB* were slightly wider than ∆*mepS* (1.20 ± 0.1 µm, 1.19 ± 0.2 µm, and 1.12 ± 0.1 µm, respectively), and ∆*amiA*∆*mrcB* and ∆*amiB*∆*mrcB* cells were shorter (2.7 ± 0.9 µm and 2.6 ± 0.8 µm, respectively) than ∆*amiA* (5.4 ± 1.3 µm) and ∆*amiB* (4.9 ± 1.0 µm). In addition, ∆*mepA*∆*mrcA* (length 4.9 ± 3.4 µm) and ∆*mepA*∆*mrcB* (6.1 ± 4.4 µm) grew longer in contrast to ∆*mepA* (4.4 ± 1.7 µm) and had an increased rate of filamentation (10.3%, 12.8%, and 5.8%). These observations point to altered functionalities of the class A PBPs in the absence of certain hydrolases but did not uncover a clear dependency of PBP1A or PBP1B on one of the tested hydrolases.

**Fig 1:**
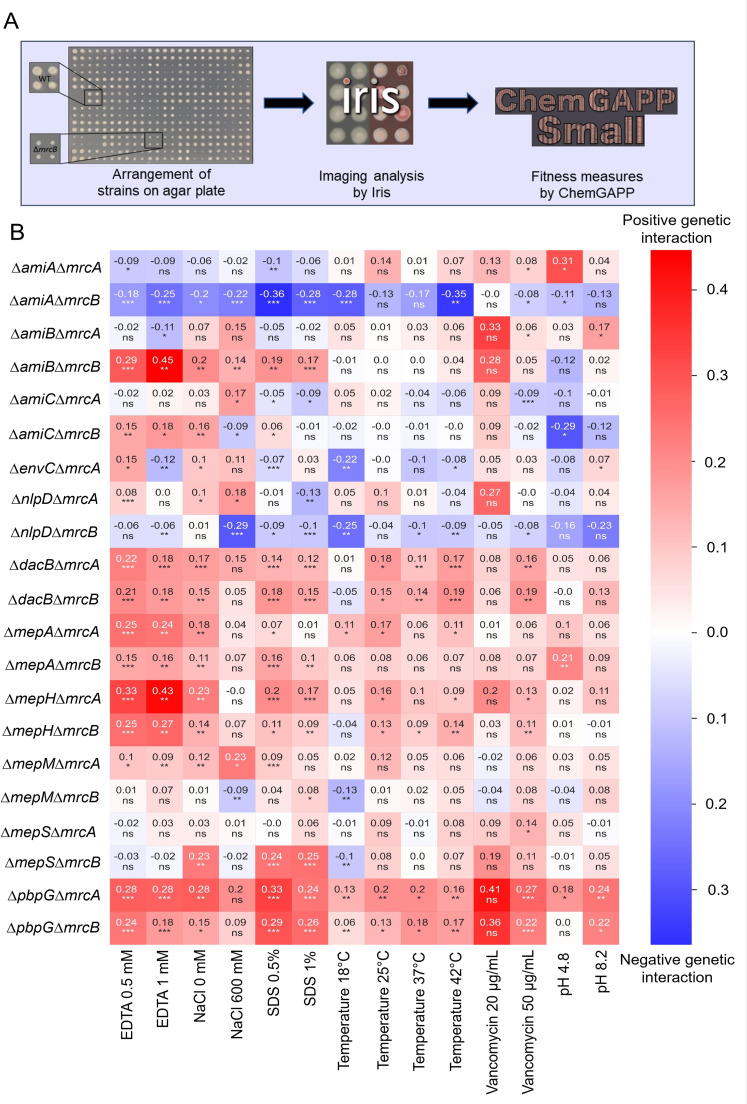
Chemical genetics screen indicates high and no NaCl conditions as the harshest stresses for the mutants tested. (**A**) Schematic of chemical genetics screening, imaging analysis of colony size, and fitness measures. The example plate shows four replicates of WT and Δ*mrcB*, illustrating the difference in the size of colony on an LB agar plate with 600 mM NaCl. (**B**) Heatmap of genetic interaction scores generated by ChemGAPP GI after chemical genomics screening of Δ*mrcA,* Δ*mrcB,* and ΔEPases (*mepS, mepA, mepH, mepM, pbpG,* and dacB) and Δamidases/regulators (*amiA, amiB, amiC, nlpD,* and *envC*) mutants under different growth conditions. The colony size was considered fitness readout (see text for details). The different colors represent the genetic interaction scores ranging from blue (negative values) to red (positive values).

This result might not be unexpected as *E. coli* harbors an arsenal of hydrolases that are collectively able to complement each other under standard laboratory conditions (37). However, previous work showed that PBP1A and PBP1B have different pH requirements to be functional (16), suggesting that PG enzymes might have specialized roles depending on the growth environment. Hence, we tested whether envelope targeting stresses impact the fitness of the mutant panel and whether the mutants genetically interact under these conditions. Mutants were pinned on agar plates supplemented with EDTA (ethylene diamine tetra acetic acid), SDS (sodium-dodecyl sulfate), vancomycin, 600 mM NaCl, 0 mM NaCl, at pH 4.8 or pH 8.2, and incubated for 18 h at 37°C or, for some conditions, at 18°C, 25°C, and 42°C to probe for heat stress. End point images were analyzed by the software Iris (40) to quantify colony size as a proxy for fitness. Fitness ratios of 1.0 indicate WT-level fitness. Values below 1.0 represent reduced fitness, and values above 1.0 indicate improved fitness of a mutant compared to WT growth in the same condition (Fig. S3). Subsequently, genetic interaction scores were calculated by ChemGAPP GI (41). The package calculates the fitness ratio of two single mutants (ΔA and ΔB) and the double mutant ΔAΔB compared to the WT strain and then calculates the expected double mutant fitness ratio, comparing it to the observed fitness ratio (Fig. 1A).

As anticipated, the envelope stress screen revealed multiple phenotypes for us to explore. Overall, the strongest fitness defects were observed in mutants lacking *mrcB* when exposed to acidic pH, EDTA, and salt stress. This is consistent with PBP1B contributing to overall fitness more than PBP1A (16).

As acidic pH severely affects *mrcB* mutants, we first looked at this condition in our panel (Fig. 1B). At pH 4.8, we found a positive genetic interaction score (0.31) when deleting both, *amiA* and *mrcA*, meaning that deletion of both genes leads to increased fitness. This could be due to the previously suggested specialized function of AmiB at acidic pH (32) promoting cell growth in the absence of AmiA and PBP1A. In contrast, we observed a negative genetic interaction score between *amiC* and *mrcB,* as deleting both genes led to decreased fitness (−0.29). We also observed a negative genetic interaction score between *amiA* and *mrcB* (−0.11). Our data suggest that a further deletion of *amiA* or *amiC* exacerbates the sickness of ∆*mrcB* cells at acidic pH (16).

The addition of the metal chelator EDTA into the media severely affected the fitness (<0.2) of all strains lacking *mepS* and the ∆*nlpD*∆*mrcB* and ∆*amiA*∆*mrcB* double mutants (Fig. S3). The depletion of MepS and MepM together is synthetically lethal (34), and EDTA abolishes the activity of MepM, a metal-dependent EPase, *in vitro* (34). This suggests that the reduced fitness of all strains lacking *mepS* in the presence of EDTA is due to the inhibition of MepM by EDTA, in agreement with a previous report (42). The severe fitness loss of ∆*amiA*∆*mrcB* is likely unrelated to MepM, as an ∆*amiA*∆*mrcB*∆*mepM* mutant is viable (Fig. S4). We observed a strong positive genetic interaction score at both EDTA concentrations (0.5mM and 1mM) of ∆*amiB*∆*mrcB* (0.29 and 0.45) indicating that inactivation of MepM is beneficial in the absence of AmiB and PBP1B. We also observed positive genetic interaction scores between ∆*mepH* and ∆*mrcA* (0.33 and 0.43) and, to a lesser extent, between ∆*mepH* and ∆*mrcB* (0.25 and 0.27) (Fig. 1B). In these cases, MepS may function better with either PBP in the absence of MepM and MepH. However, as EDTA causes pleiotropic effects on both cell membranes beyond the inactivation of MepM, it is difficult to draw more detailed conclusions.

Salt (NaCl) stress modulates the intracellular osmotic pressure by either decreasing it (high salt levels) or increasing it (low salt levels). Both osmotic extremes affect the envelope integrity negatively as they potentially constrain membrane fluidity, protein-protein interactions, and protein function. We noticed that the ∆*mrcB* single mutant had decreased fitness (0.49 and 0.38) at both, 0 and 600 mM NaCl (Fig. S3), resulting in many positive and negative genetic interaction scores (Fig. 1B). Most notably, in 600 mM NaCl, we observed genetic interaction scores of *mrcB* with *amiA* (−0.22), *amiB* (0.14), *nlpD* (−0.29), and *amiC* (−0.09). As NlpD activates AmiC, it is expected that both genetic interactions phenocopy each other. Interestingly, we observed a negative genetic interaction score for *mrcB* with *amiA* in 600 mM NaCl, but not for *mrcB* and *amiB*, despite both amidases sharing EnvC as their activator, suggesting that AmiB becomes important at high NaCl concentrations.

The envelope stress screen of the PBP-hydrolase mutant panel confirmed several of the already known genetic interaction pairs in pH and EDTA stress. In addition, we observed that ∆*mrcB* had reduced fitness in salt stress indicating that PBP1A alone might not be sufficient to uphold cell growth. To test this hypothesis, we next explored how salt stress affects the morphology of some of the combined *mrcB* and hydrolase mutants.

### *E. coli* needs *mrcB* or *amiC-nlpD* under high salt stress

To test how salt stress affects the cell morphology, we analyzed some of the most severely affected mutants: ∆*amiC*∆*mrcB*, ∆*nlpD*∆*mrcB,* ∆*mepS*∆*mrcB,* and the respective control strains, using phase contrast and fluorescence microscopy as described above.

**Fig 2:**
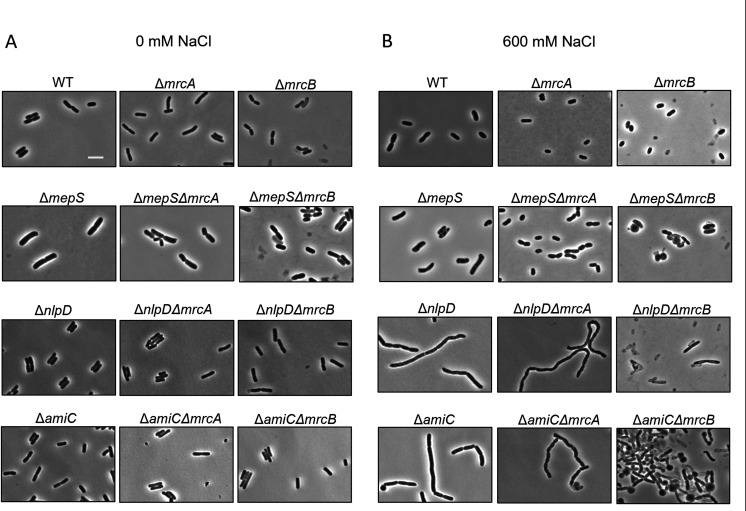
Deletion of *mepS, nlpD,* or *amiC* enhances the growth defect of Δ*mrcB* mutants under high salt stress. Phase contrast microscopy of cells of WT and different mutants in the exponential phase (OD_600_≅ 0.2) back-diluted to media altered with (**A**) 0 mM NaCl or (**B**) 600 mM NaCl and grown until OD_600_≅ 0.2. Scale bar = 5 µm. Scale bar shown is representative for all images. Images of fluorescence microscopy are shown in Fig. S5.

As expected, ∆*mrcB* cells were sick in both salt stresses, and 16% of all analyzed ∆*mrcB* cells showed signs of cell lysis (Fig. 2; Fig. S5; Table S3). In 600 mM NaCl, the lysis of ∆*mepS*∆*mrcB* was increased to 40% (Fig. 2; Fig. S5; Table S3). Considering the prominent role of MepS in cell elongation and its interaction with PBP1A (21, 34), the increased lysis of ∆*mepS*∆*mrcB* in high salt stress is not surprising. Almost all cells of ∆*amiC*∆*mrcB* and ∆*nlpD*∆*mrcB* showed signs of lysis confirming the observed severe negative genetic interactions (Fig. 1). However, ∆*amiC*∆*mrcA* and ∆*nlpD*∆*mrcA* cells exclusively chained (>95% of all cells) compared to their controls, ∆*mrcA* (0%), ∆*amiC* (49.2%), and ∆*nlpD* (65.5%) (Fig. 2; Fig. S5; Table S3). As both ∆*amiC*∆*mrcA* and ∆*nlpD*∆*mrcA* also had positive genetic interactions, this indicates that in high salt, cells need either *mrcB* or *nlpD-amiC* to successfully separate into daughter cells. In LB containing no NaCl, we did not observe notable morphological abnormalities in the strains tested though some lysis was visible for ∆*mrcB*, ∆*nlpD*∆*mrcB,* and ∆*mepS*∆*mrcB* (Fig. 2A; Fig. S5; Table S3). Together, these data provide additional evidence that the observed phenotypes may be related to the inability of class A PBPs to properly function in cells growing in the presence of high concentration of NaCl.

### Normal PG cross-linkage in ∆*mrcA* and ∆*mrcB* cells

As we had observed morphological defects in some of the mutants growing at high salt concentration, presumably due to reduced class A PBP activity, we asked whether the PG produced in the ∆*mrcA* and ∆*mrcB* strains is more fragile due to reduced cross-linkage. To address this question, we first monitored the growth of the mutants in the LB medium with two different NaCl concentrations, 350 and 500 mM, to choose the best growth condition for PG analysis (Fig. S6A and B). At 500 mM NaCl, both ∆*mrcA* and ∆*mrcB* strains had a longer lag time and a longer doubling time than the WT (Fig. S6B) and both suffered from extensive lysis (Fig. S6D). Interestingly, cells of both mutants formed bulges at the cell periphery before bursting, similar to those observed in β-lactam-induced lysis of wild-type cells, providing a further hint that ∆*mrcA* and ∆*mrcB* cells might suffer from insufficient d,d-TPase activity when grown at high salt concentration. At 500mM salt, the WT cells showed cell branching, indicating defects in division ring placement (Fig. S6D, left panel); this phenotype is similar to that of mutants lacking multiple d,d-carboxypeptidases and PBP1A (43). At 350 mM NaCl, the growth curve of ∆*mrcA* was similar to the WT, while ∆*mrcB* showed a longer lag time than the WT, and the doubling times were similar for the three strains (Fig. S6A). Lysed cells were also visible at 350mM NaCl for ∆*mrcA* and ∆*mrcB* but were less frequently than at 500 mM NaCl (Fig. S6C). These results also confirm that the growth defects for ∆*mrcB* at high salt are more severe than for ∆*mrcA*.

**Fig 3:**
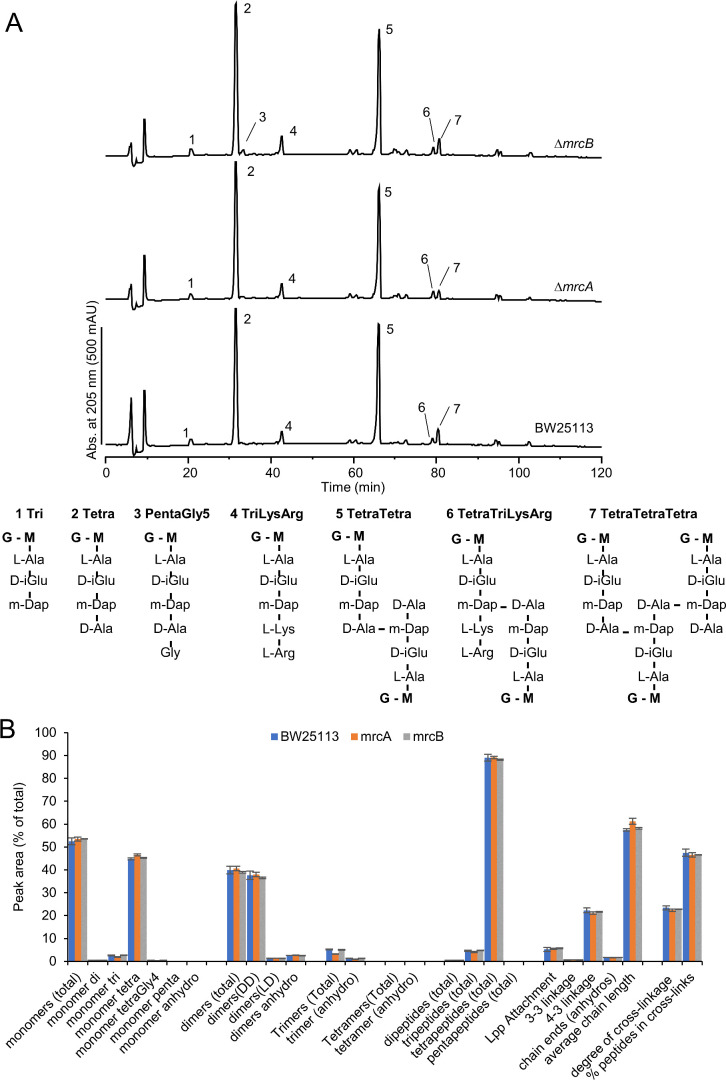
PG from ∆*mrcA* and ∆*mrcB* grown at 350 mM NaCl is similar to PG from WT. BW25113 (WT), BW25113∆*mrcA*::kan (∆*mrcA*), and BW25113∆*mrcB*::kan (∆*mrcB*) were grown in duplicate in LB containing 350 mM NaCl, and cells were harvested at an OD_600_ of 0.7–0.8. PG was isolated and digested with cellosyl, and the resulting muropeptides were analyzed by HPLC. (**A**) Chromatograms showing the muropeptide peaks from PG isolated from one of the duplicates of WT, ∆*mrcA,* and ∆*mrcB*. The main peaks are labeled, and their structures are shown on the right side. (**B**) Analysis of the peak areas from chromatograms showing the peak areas if muropeptides from WT (blue), ∆*mrcA* (orange), and ∆*mrcB* (gray). Error bars, mean ± variation of two biological replicates.

We next isolated the PG and determined the muropeptide composition of cells grown at 350 mM NaCl to an optical density of 0.7–0.8 (Fig. 3A). At this time point, there were differences in growth between the WT and the mutants, but there was no extensive lysis, which could interfere with PG isolation. The analysis of the PG from BW25113 and the ∆*mrcA* and ∆*mrcB* mutants showed only modest differences between the three strains (Fig. 3B), suggesting that the fragility of these mutants at high salt is not due to reduced PG crosslinking.

### The activity of class A PBPs is reduced at high salt

To determine if the activities of PBP1A and PBP1B are affected by NaCl, we assayed the purified enzymes in two buffer conditions with different concentrations of NaCl: low salt (minimum NaCl concentration achievable upon dilution of proteins stock into reaction conditions, 30 mM NaCl for PBP1B and 45 mM for PBP1A) and high salt (500 mM NaCl). We used three different activity assays to monitor the glycosyltransferase and/or transpeptidase activities of these proteins. In every case, we measured the activity of PBP1A and PBP1B in the presence or absence of their cognate activator, LpoA and LpoB, respectively.

**Fig 4:**
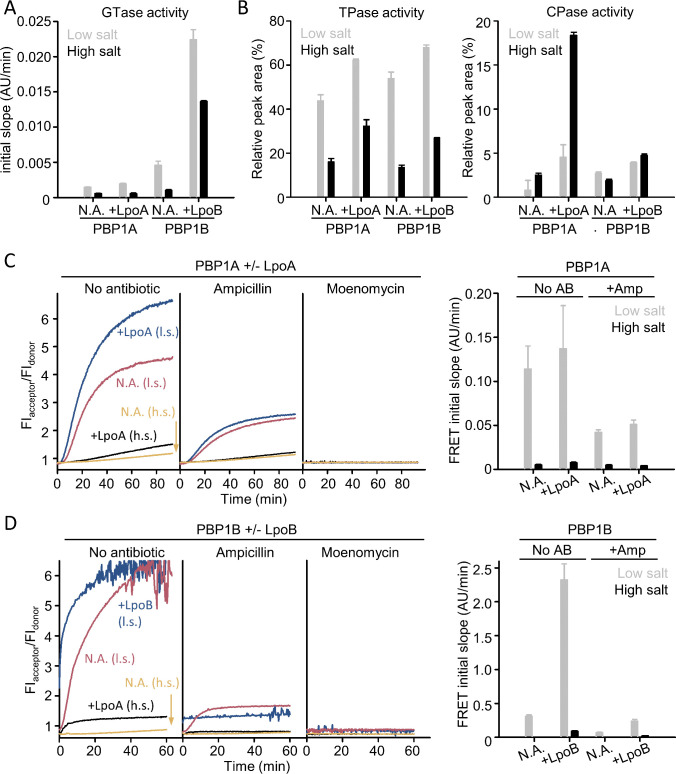
The activities of PBP1A and PBP1B are reduced at high salt concentrations. The activity of PBP1A and PBP1B was tested by three different assays in low (30 and 45 mM NaCl for PBP1A and PBP1B, respectively) or high (500 mM NaCl) salt concentrations and in the presence (+LpoA or +LpoB) or absence (N.A.) of activators. (**A**) GTase assay with dansyl-lipid II. Initial slopes were obtained with 0.5 µM PBP1A or PBP1B and 10 µM dansyl-lipid II at low (gray) and high salt (black) conditions. Samples were incubated for 90 min at 30°C (PBP1A) or 30 min at 25°C (PBP1B). Values are mean ± SD for three repeats. (**B**) End-point assay with radiolabeled lipid II. Relative peak area for TPase products (left, peaks 5–10 in Fig. S7C and D) or CPase products (right, peaks 2 and 5 in Fig. S7C and D) for reactions with 0.5 µM PBP1A or 0.7 µM PBP1B and 25 µM [^14^C]-lipid II at low (gray) and high (black) salt. Values are mean ± variation for two repeats. (**C**) Continuous FRET-assay with PBP1A and unlabeled and fluorescently labeled lipid II. Left side: representative reaction curves from PBP1A at low (blue with LpoA, red without activator) and high salt conditions (black with LpoA, yellow without activator), respectively. Right side: initial slopes calculated from FRET curves obtained from PBP1A assays at low (gray) and high salt (black). Values are mean ± SD (or variation) of at least two repeats. (**D**) Continuous FRET-assay with PBP1B. Left side: representative reaction curves from FRET assays of PBP1B at low (blue with LpoB, red without activator) and high salt conditions (black with LpoB, yellow without activator), respectively. Right side: initial slopes calculated from FRET curves obtained from PBP1B assays at low (gray) and high salt (black). Values are mean ± SD or variation of at least two repeats. In (**C**) and (**D**), ampicillin (1 mM) or moenomycin (50 µM) were added when indicated and reactions. The concentration of Lpo activators was four times the one of their cognate enzyme.

We first used a real-time GTase assay using dansyl-labeled lipid II which is based on the reduction in fluorescence intensity of the dansyl probe upon polymerization of glycan strands and subsequent digestion to muropeptides by cellosyl present in the reaction (Fig. 4A; Fig. S7A and B) (44). The apparent GTase rate obtained in this assay for PBP1A reactions was 60%–70% lower at high salt than at low salt (Fig. 4A; Table 1). LpoA mildly activated the PBP1A GTase rate at low salt and had no effect at high salt (Fig. 4A; Table 1). PBP1B had ~80% lower apparent GTase activity at high salt than at low salt. LpoB strongly increased the GTase rate, as previously published (4, 45, 46), but at high salt, the apparent GTase rate with activator was ~40% decreased compared to low salt (Fig. 4A; Table 1). Interestingly, the apparent GTase rates obtained for PBP1B were nearly an order of magnitude higher than those for PBP1A when comparing reactions under the same buffer conditions (Fig. 4A; Table 1). This observation is even more relevant considering that PBP1B reactions were performed at 25°C and PBP1A reactions at 30°C.

**TABLE 1:** Relative GTase and TPase rates of PBP1A and PBP1B, and % TPase/CPase products in the PG produced, at different reaction conditions

Condition	Enzyme/activator	GTase rate (dansyl-LII)^*a*^	GTase rate (FRET)^*b*^	GTase + TPase rate (FRET)^*b*^	% TPase products^*c*^	% CPase products^*c*^
Low salt	PBP1A	32 ± 1	65 ± 5	37 ± 8	44% ± 3%	1% ± 1%
	PBP1A + LpoA	42 ± 2	78 ± 8	44 ± 16	62.3% ± 0.5%	5% ± 1%
High salt	PBP1A	12 ± 1	7 ± 1	1.7 ± 0.2	16% ± 1%	2.5% ± 0.2%
	PBP1A + LpoA	12 ± 3	6.1 ± 0.5	2.5 ± 0.2	32% ± 3%	18.4% ± 0.3%
Low salt	PBP1B	100 ± 13	100 ± 22	100 ± 8	54% ± 3%	2.7% ± 0.2%
	PBP1B + LpoB	492 ± 32	365 ± 43	752 ± 76	68% ± 1%	3.9% ± 0.1%
High salt	PBP1B	22 ± 3	1.6 ± 0.4	0.8 ± 0.2	13.7% ± 0.9%	1.9% ± 0.2%
	PBP1B + LpoB	300 ± 2	31 ± 3	29 ± 2	26.9% ± 0.1%	4.7% ± 0.2%

^*a*^ GTase rates with dansyl-lipid II (Fig. 4A), normalized to the rate of PBP1B at low salt and no activator.

^*b*^ GTase and combined GTase + TPase rates were obtained from FRET assays (Fig. 4C and D) in the presence and absence of ampicillin, respectively. Rates are normalized to the rate of PBP1B at low salt and no activator.

^*c*^ Percentage of radioactivity in TPase and CPase products, obtained in assays with [^14^C]-lipid II (Fig. 4B).

We next analyzed the TPase function of both PBPs in the same conditions as above using an end-point assay in which the PG product from radiolabeled lipid II was digested with the muramidase cellosyl, which cleaves glycan strands between *N*-acetylmuramic and *N*-acetylglucosamine residues, and the resulting muropeptides were quantified by HPLC (Fig. 4B and C; Fig. S7C and D) (19, 47). The percentage of transpeptidation products decreased at high salt compared to low salt conditions for both proteins, with a higher decrease for PBP1B than for PBP1A (Fig. 4B, left panel). LpoA and LpoB increased the proportion of transpeptidation products for their cognate enzyme in both conditions (Fig. 4B, left panel). Interestingly, we noticed a substantial increase in d,d-carboxypeptidase (CPase) products for PBP1A in the presence of LpoA at high salt compared to low salt conditions without LpoA (Fig. 4B, right panel); such an increase was not observed for PBP1B (Fig. 4B, right panel). We noticed previously that both PBP1A and PBP1B have inherent CPase side activity, which is enhanced at acidic pH in the presence of the cognate activator, removing the terminal d-Ala at position 5 of the donor peptide instead of performing a TPase reaction (3). As this is an end-point assay that shows the composition of the PG product synthesized, it was not possible to distinguish differences in the TPase rates between PBP1A and PBP1B. Nonetheless, these results show that PBP1B produced a slightly more crosslinked PG (54% ± 3% cross-linked peptides) than PBP1A (44% ± 3% cross-linked peptides) at low salt and PBP1A produced a slightly more cross-linked PG (16% ± 1% cross-linked peptides) than PBPB (13.7% ± 0.9% cross-linked peptides) at high salt (Fig. 4B; Table 1).

Finally, we used a recently developed FRET-based assay employing two types of labeled lipid II (lipid II-Atto550 and lipid II-Atto647n) in the presence of unlabeled lipid II that can monitor both, GTase and TPase activities in real time (Fig. 4C and D) (48). We used this assay previously to monitor the GTase and TPase activities of PBP1B homologs from *E. coli*, *Pseudomonas aeruginosa,* and *Acinetobacter baumannii*. In this assay, the apparent GTase rates are obtained when adding ampicillin to block the TPase. In the absence of ampicillin, the combined apparent GTase+ TPase rates are obtained, as crosslinking increases the FRET by bringing more probes from different glycan strands close together. After having followed FRET development over time by measuring fluorescence emission ratio of donor and acceptor at specific wavelengths, we recorded spectra to measure the final amount of FRET developed, which indicates the activity level, and the products were analyzed by SDS-PAGE to confirm the presence of crosslinked PG and the consumption of labeled lipid II. To interpret the results, it was also important to determine whether the class A PBP can polymerize labeled lipid II in the absence of unlabeled lipid II. We previously reported that only LpoB-activated PBP1B can utilize the labeled lipid II versions in the absence of unlabeled lipid II (48). Here, we found that PBP1A was unable to polymerize labeled lipid II in the absence of unlabeled lipid II, irrespective of the presence or absence of LpoA (Fig. S8). This simplified the interpretation of FRET signals in the presence of LpoA, as any increase in FRET, when LpoA is present, will be due to stimulation of GTase or TPase rates and not due to probes being incorporated closer on the same glycan strands. Finally, we also verified that fluorescence intensities of lipid II-Atto550 and lipid II-Atto647n were not affected by the increase in ionic strength at high salt (Fig. S9).

When we performed FRET assays including unlabeled lipid II in high salt condition, both PBP1A and PBP1B showed a substantially smaller amount of FRET at the end of the reaction (Fig. S10 and S11), a reduced consumption of lipid II, and less production of PG chains or crosslinked PG, compared to low salt conditions (Fig. S12). In addition, the apparent GTase and GTase+ TPase rates were~15–20 times smaller at high salt than at low salt for both enzymes, with or without activator (Fig. 4C and D; Table 1). PBP1B consumed more lipid II, produced higher final FRET, and showed higher GTase or GTase+ TPase rates than PBP1A in every condition tested (Fig. 4C and D; Fig. S10 to S12), in agreement with the results from the dansyl-lipid II assay (Fig. 4A; Fig. S7A and B). The addition of LpoA slightly increased the apparent GTase and GTase+ TPase rates at low salt but had no effect on the GTase rate of PBP1A at high salt (Fig. 4C; Table 1). By contrast, LpoB activated PBP1B robustly at both high and low salt conditions (Fig. 4D; Table 1).

In summary, the GTase and TPase activities of both class A PBPs were considerably reduced at high salt. The end-point TPase assay showed a modest reduction in the degree of crosslinking of the PG produced by both enzymes at high salt, which, together with the strongly reduced GTase+ TPaserate in the FRET assay, is indicative of a reduced TPase reaction rate at high salt, as discussed in reference (48). The real-time assays revealed that PBP1B is up to one order of magnitude more active than PBP1A in similar conditions (Table 1). LpoB activated both, GTase and TPase of PBP1B at low and high salt conditions, but LpoA had only a modest effect on the GTase and GTase+ TPase of PBP1A at low salt conditions and only slightly activated the GTase+ TPaserate, but not the GTase rate, at high salt.

## DISCUSSION

Two decades ago, Höltje proposed that the expansion of the PG sacculus requires the collaborative action of synthases and hydrolases (49). This concept, along with other works, supports that PG multi-protein complexes are dynamic and driven by protein-protein interactions (37). Recent work gained some understanding of the redundancy of PG hydrolases, showing that some of these enzymes might be more specialized to act in specific environmental stresses (16, 38, 50–52). Here, we explored the potential connections of *E. coli* PG hydrolases with the two major class A PBPs, PBP1A and PBP1B, in various environmental conditions. This genetic PBP-hydrolase interaction network did not uncover strong PBP-hydrolase genetic interaction pairs that would link specific hydrolases to PBP1A or PBP1B. However, we uncovered that the fitness of ∆*mrcB* cells is significantly reduced under salt stress. This observation is consistent with the recent findings that outer membrane-anchored LpoB regulates PBP1B in response to osmotic challenges (upon upshift in salt or sucrose concentration). This regulation is partly lost in cells with cytoplasmic membrane-mislocalized LpoB, which have reduced sickness in high salt (4, 53), demonstrating that the LpoB-mediated regulation of PBP1B is responsive to media osmolarity and salt concentration. This led us to investigate the effects of salt on the activities of purified PBP1A and PBP1B.

We found that, *in vitro*, at high salt, both PBP1A and PBP1B produced PG with reduced crosslinking compared to low salt conditions, where the enzymes were fully active. The results suggest that a low residual class A PBP activity could be the reason for morphological defects observed in mutants lacking one of the class A PBPs at high salt, while no morphological changes are observed at low salt. Interestingly, an *mrcB* mutant strain showed more severe growth defects than the *mrcA* mutant, which required higher NaCl concentration for impaired growth (Fig. S6A and B). As PBP1A had lower *in vitro* PG synthesis rates than PBP1B, including at high salt, it is possible that PBP1A alone is unable to supply the necessary class A PBP activity required for survival. Indeed, PBP1B had faster activity rates *in vitro* than PBP1A in every assay and condition, in agreement with previous reports (3, 54) and, importantly, LpoB was still capable of activating PBP1B at high salt, while LpoA did not activate PBP1A (GTase) at high salt.

We also analyzed the PG composition in mutant strains lacking one of the class A PBPs in conditions that affected the growth but did not cause extensive lysis. The PG composition was similar in the mutants experiencing mild salt stress (Fig. S6B), suggesting that cells are still capable of producing a PG with normal composition. Therefore, we hypothesize that the reason for the lysis of these strains at high salt is an insufficient PG synthesis capacity, which cannot be compensated by other PG synthases like SEDS-class B PBPs and the monofunctional GTase MtgA. Consistent with this hypothesis, Δ*mrcB* cells develop bulges and lyse at high salt in a similar way as WT cells at standard condition upon inhibition of PG synthesis by antibiotics. *E. coli* cells produce a dynamic PG triangular wedge at the lagging edge of the septum, which appears to be important for robustness of the septum; the thickness of this wedge might depend on the balance between PG synthesis and hydrolysis rates (55). Class A PBPs were hypothesized to produce the PG in this wedge (55), consistent with earlier work showing that PBP1A and PBP1B interact with the membrane anchors of FtsZ, ZipA (directly), and FtsA (via FtsN), to localize at pre-septal sites before cell division starts (56). A high salt concentration might impair the robustness of this pre-septal PG synthesis due to the reduced activity of class A PBPs, thereby disturbing the balance between the synthesis and hydrolysis of PG and causing a more fragile PG during septum initiation which could lead to cell lysis. Interestingly, all tested stains, including the WT, show morphological changes at high salt, most strikingly cell branching (Fig. S6D). This might indicate a less robust cell division placement as has been observed before in strains lacking multiple CPases and PBP1A (43).

It has been recently suggested that LpoA is necessary to stimulate the GTase activity of PBP1A as mutations that confer higher GTase activity *in vitro* allow the deletion of *lpoA in vivo* (57). However, the study provided no direct evidence for an effect of LpoA on the GTase activity of PBP1A. Here, we observed only a mild stimulation of PBP1A’s GTase by LpoA at low salt, and no activation at high salt, while the TPase was activated in both conditions. Thus, our results confirm previous reports indicating that the main effect of LpoA is the activation of the TPase (46, 58).

## MATERIALS AND METHODS

### Bacterial strains and growth conditions

In this study, we used *E. coli* BW25113 as the parental strain. *E. coli mrcA::tet, mrcB::tet, dacB::kan, pbpG::kan, mepS::kan, mepM::kan, mepA::kan, mepH::kan, amiA::kan*, *amiB::kan, amiC::kan, nlpD::kan,* and *envC::kan* mutants were obtained from the KEIO collection (59) and transduced into a new parental strain. The double knockouts were created by P1 transduction as previously described (15, 60). The chromosomal modification was verified by colony PCR to detect the Kan^R^ and Tet^R^ cassette. Strains were grown on LB agar (5 g/L NaCl, 5 g/L yeast extract, 10 g/L tryptone, 1.5% agar) and LB broth (5 g/L NaCl, 5 g/L yeast extract, 10 g/L tryptone). Table S1 shows the bacterial strains used in this study.

### Chemical genetic screening

For each strain probed, a single source plate was generated and transferred to the final screening plate using a pinning robot (Biomatrix 6). Chemical perturbations (1% SDS; 0.25 mM and 0.5 mM EDTA; 0.25% SDS and 0.25 mM EDTA; pH 4.8; pH 8.2; 0 mM NaCl; 600 mM NaCl; 50 µg/mL vancomycin) were added to the LB agar or adjusted before dispensing into the plates. MMT buffer (1:2:2 molar ratio of d,l-malic acid, MES, and Tris base) was added to the media adjusted to pH 4.8 and 30 mM HEPES-KOH buffer to pH 8.2. On each screening assay plate, the parental strain, the single mutants, and the double mutants were arrayed, each in approximately 20 replicates per plate. The plates were incubated at 37°C (or for three conditions at 18°C, 25°C, and 42°C) for ~18 h and imaged under controlled lighting conditions (S&P Robotics) using an 18-megapixel Canon Rebel T3i (Canon). Colony size of endpoint images was quantified as fitness readout using the image analysis software Iris. Fitness ratio and statistical analysis were carried out by ChemGAPP as described below.

### ChemGAPP analysis

Colony size data for each plate were checked for plate effects via a Wilcoxon rank sum test between the distributions of colony sizes for the outer two edges of the plate and the center colonies. For *P* < 0.05, the difference in the distributions was considered statistically significant, and the outer edge colonies were scaled to the plate middle mean (PMM). The plate middle mean is defined as the mean colony size of colonies within the center of the plate, within the 40th and 60th percentile of size. Each plate was then scaled such that the PMM was equal to the median colony size of all colonies within the screen. Significance values were denoted as such: *: 1.00e−02 < *P* ≤ 5.00e−02; **: 1.00e−03 < *P* ≤ 1.00e−02; ***: 1.00e−04 < *P* ≤ 1.00e−03; ****: *P* ≤ 1.00e−04.

For determining genetic interaction scores, for each condition, replicate plate, and gene pair, the double expected fitness ratio was calculated by multiplying the two single knockout fitness ratios produced by ChemGAPP Small. The genetic interaction score was then calculated for each gene pair by subtracting the double expected fitness ratio from the double observed fitness ratio. Replicate genetic interaction scores from the same condition were averaged for each gene pair and presented in a heatmap. Positive scores represented a positive genetic interaction and negative scores represented a negative genetic interaction. Significance was calculated via a one-way ANOVA between the replicate double expected fitness ratios and the replicate double observed fitness ratios.

### Microscopy

FM1-43FX (Invitrogen) dye was used for membrane visualization, and DAPI (Stratech Scientific) was used to stain DNA for fluorescence microscopy. Overnight cultures were diluted into LB broth to an OD_600_ = 0.01 and incubated at 37°C until mid-exponential phase (OD_600_ = ~0.4). Next, 0.5 mL of the culture wase stained with 5 µg/mL of FM1-43FX and incubated at room temperature for 10 min. The cells were then brought into 33 mM sodium phosphate pH 7.4 and fixed with 2.4% formaldehyde and 0.04% glutaraldehyde. The cells were washed several times in PBS to remove the excess of reagents. For microscopy visualization, the cells were placed on 1.5% agarose pads set in Gene Frames (Thermos Scientific). Cells were imaged by Zeiss AxioObserver microscope under Plan-Apochromat 100×/Oil Ph3 objective and illumination from HXP 120 V for phase contrast images. FM1-43FX images were visualized by Zeiss filter set 38 (Ex: 470/40 nm, beam splitter 495 nm, Em: 525/50 nm). For DAPI visualization, Zeiss filter set 96 (Ex: 390/40 nm, beam splitter 420 nm, Em: 450/40 nm) was used. Phenotype analyses were generated by MicrobeJ plugin for Fiji.

For quantifying the extent of cell lysis in a culture, we determined the fraction of phase light cells manually, as these were not detected by MicrobeJ. Tables S2 and S3 depict the total number of cells as the sum of viable cells (detected by MicrobeJ) and lysed cells (not detected by MicrobeJ).

Imaging of cells grown in stress conditions: the cultures were grown at 37°C in standard LB broth (pH 6.9) overnight. From this culture, cells were grown at 37°C in LB medium to early exponential phase (OD_600_ ≅ 0.2), back-diluted to media containing either 600 mM NaCl or 0 mM NaCl, and grown until OD_600_ ≅ 0.2. Microscopy slide preparation and data analysis were conducted as described above.

In this study, we conducted a comparative analysis between mutant and WT strains of cell length, cell width, and the number of septa. To rigorously assess differences, we employed a Student’s two-sample *t*-test, specifically the Welch two-sample *t*-test, performed using the R statistical software (version 4.3.1), for each mutant strain in comparison to the WT data.

### Purification of proteins

The following *E. coli* proteins were purified following published protocols: PBP1B (15), LpoB(sol) (45), PBP1A (61), and LpoA(sol) (62). All chromatographic steps were performed using an AKTA PrimePlus system (GE Healthcare).

PBP1A was expressed from plasmid pTK1A in *E. coli* LOBSTR strain (63), as a fusion with an N-terminal oligohistidine tag. Cells were grown in 4 L of LB plus autoinduction media (0.5% glycerol, 0.05% glucose, 0.2% lactose) supplemented with kanamycin at 30°C for ~16 h. Cells were pelleted by centrifugation (10,000  ×  *g*, 15  min, 4°C) and resuspended in 80  mL of buffer I (25 mM Tris-HCl, 100 mM NaCl, 10 mM MgCl_2_, 10% glycerol, pH 7.5) supplemented with 1 × protease inhibitor cocktail, 100 µM PMSF, and Dnase I. After disruption by sonication on ice, membrane fraction was pelleted by centrifugation (130,000  ×  *g* for 1  h at 4°C) and washed with 80 mL of buffer II (buffer I with 1 M NaCl instead) by stirring for 2 h. After centrifugation (130,000  ×  *g* for 1  h at 4°C), the membrane pelleted was solubilized by stirring in buffer II plus 2% Triton X-100 for 16 h at 4°C. Extracted membranes were separated from insoluble debris by centrifugation (130,000  ×  *g* for 1  h at 4°C) and incubated for 2 h with 4 mL of Ni^2+^-NTA beads (Novagen) equilibrated in buffer II plus 0.2% Triton X-100. Beads were washed 10 times with 10 mL of buffer II plus 0.2% Triton X-100 and 50 mM imidazole, and the protein was eluted with 3 mL Elution buffer (buffer II plus 0.2% Triton X-100 and 500 mM imidazole). His-PBP1A-containing fractions were pooled and treated with 2 U/mL of thrombin (Novagen) for 20 h at 4°C during dialysis against dialysis buffer I. Protein was then dialyzed in preparation for ion exchange chromatography, first against dialysis buffer A (20 mM sodium acetate, 0.5 M NaCl, 10% glycerol, pH 4.8) and then against dialysis buffer B (20 mM sodium acetate, 0.2 M NaCl, 10% glycerol, pH 4.8). Finally, the sample was applied to a 1 mL HiTrap SP column (GE Healthcare) equilibrated in buffer IEX-A (20 mM sodium acetate, 100 mM NaCl, 10% glycerol, 10 mM MgCl_2_, 0.1% reduced Triton X-100, pH 4.8), using an AKTA Prime system. The protein was eluted with a gradient from 0% to 100% buffer B (as A, with 2 M NaCl) over 14 mL. PBP1A-containing fractions were pooled and dialyzed against storage buffer (25 mM Hepes, 300 mM NaCl, 10% glycerol, pH 7.5) and stored at −80°C.

PBP1B was expressed as a fusion with an N-terminal hexahistidine tag in *E. coli* BL21(DE3) pDML924 grown in 4 L of autoinduction medium (LB medium supplemented with 0.5% glycerol, 0.05% glucose, and 0.2% α-lactose) containing kanamycin at 30°C for ~16 h. Cells were harvested by centrifugation (10,000  ×  *g*, 15  min, 4°C) and the pellet resuspended in 80 mL of buffer I (25 mM Tris-HCl, 1 M NaCl, 1 mM EGTA, 10% glycerol, pH 7.5) supplemented with 1 × protease inhibitor cocktail (PIC, Sigma-Aldrich), 100 µM phenylmethylsulfonyl fluoride (PMSF, Sigma-Aldrich), and Dnase I. After disruption by sonication on ice, membrane fraction was pelleted by centrifugation (130,000  ×  *g* for 1  h at 4°C) and resuspended in buffer II (25 mM Tris-HCl, 1 M NaCl, 10% glycerol, 2% Triton X-100, pH 7.5) by stirring at 4°C for 24 h. Extracted membranes were separated from insoluble debris by centrifugation (130,000  ×  *g* for 1 h at 4°C) and incubated for 2 h with 4 mL of Ni^2+^-NTA beads (Novagen) equilibrated in buffer III (25  mM Tris-HCl, 1 M NaCl, 20  mM imidazole, 10% glycerol, pH 7.5). Beads were washed 10 times with 10 mL of buffer III, and the protein was eluted with 3 mL buffer IV (25  mM Tris-HCl, 0.5 M NaCl, 20 mM imidazole, 10% glycerol, pH 7.5). His-PBP1B containing fractions were pooled and treated with 2 U/mL of thrombin (Novagen) for 20 h at 4°C during dialysis against dialysis buffer I (25 mM Tris-HCl, 0.5 M NaCl, 10% glycerol, pH 7.5). Protein was then dialyzed in preparation for ion-exchange chromatography, first against dialysis buffer II (20 mM sodium acetate, 0.5 M NaCl, 10% glycerol, pH 5.0); then against dialysis buffer II with 300 mM NaCl; and finally against dialysis buffer II with 100 mM NaCl. Finally, the sample was applied to a 1 mL HiTrap SP column (GE Healthcare) equilibrated in buffer A (20 mM sodium acetate, 100 mM NaCl, 10% glycerol, 0.05% reduced Triton X-100, pH 5.0). The protein was eluted with a gradient from 0% to 100% buffer B (as A, with 2 M NaCl) over 14 mL PBP1B-containing fractions were pooled and dialyzed against storage buffer (20 mM sodium acetate, 500 mM NaCl, 10% glycerol, pH 5.0) and stored at −80°C.

LpoA(sol) was expressed on *E. coli* BL21(DE3) transformed with pET28His-LpoA(sol). Cells were grown in 1.5 L of LB plus kanamycin at 30°C to an OD_578_ of 0.4–0.6 and expression was induced with 1 mM of IPTG for 3  h at 30°C. Cells were pelleted and resuspended in buffer I (25 mM Tris-HCl, 10 mM MgCl_2_, 500 mM NaCl, 20 mM imidazole, 10% glycerol, pH 7.5) plus Dnase, protease inhibitor cocktail (Sigma), and PMSF. Cells were disrupted by sonication on ice and centrifuged (130,000  ×  *g*, 1 h, 4°C) to remove debris. The supernatant was applied to a 5 mL HisTrap HP column (GE Healthcare) equilibrated in buffer I using an AKTA PrimePlus (GE Healthcare). After washing with buffer I, the protein was eluted with a stepwise gradient with buffer II (25 mM Tris-HCl, 10 mM MgCl_2_, 500 mM NaCl, 400 mM imidazole, 10% glycerol, pH 7.5). Fractions containing the protein were pooled, and the His-tag was removed by the addition of 2 U/mL of thrombin (Novagen) while dialyzing against buffer IEX-A (20 mM Tris-HCl, pH 8.0). Digested protein was applied to a 5 mL HiTrap Q HP column (GE Healthcare). After washing with 85% buffer IEX-A and 15% buffer IEX-B (20 mM Tris-HCl, 500  mM NaCl, pH 8.0), the protein was eluted with a linear gradient from 15% to 100% B over 150 mL. Fractions containing LpoA protein were pooled and concentrated for application to a Superdex200 HiLoad 16/600 size exclusion column using buffer SE (25 mM Tris-HCl, 10 mM MgCl_2_, 500 mM NaCl, 10% glycerol at pH 7.5) as elution buffer. Finally, the protein was dialyzed against storage buffer (25 mM HEPES-NaOH, 200 mM NaCl, 10% glycerol at pH 7.5) and stored at −80°C.

LpoB(sol) was purified from strain BL21(DE3) pET28His-LpoB(sol) following a similar protocol as described for LpoA(sol), with modifications. After the first purification step with a HisTrap HP column, fractions containing the protein were pooled, supplemented with 2 U/mL of thrombin (Novagen) to remove the His‐tag, dialyzed against 25 mM Tris-HCl, 100 mM NaCl, 10% glycerol, pH 8.3, and applied to a 5 mL HiTrap Q HP column (GE Healthcare) attached to an AKTA Prime (GE Healthcare) at 0.5 mL/min. LpoB was collected in the flow-through and concentrated and applied to size exclusion on a Superdex200 HiLoad 16/600 column at 1 mL/min in a buffer containing 25 mM HEPES-NaOH, 1 M NaCl, 10% glycerol at pH 7.5. Finally, the protein was dialyzed against storage buffer (25 mM HEPES-NaOH, 200 mM NaCl, 10% glycerol at pH 7.5) and stored at −80°C.

### PG isolation and analysis

PG was isolated from *E. coli* cells and analyzed by reversed-phase HPLC as described (64).

### Continuous glycosyltransferase assay using dansylated lipid II

Continuous fluorescence GTase assays using dansylated lipid II were performed as previously described with minor modifications (44, 65, 66). Samples contained 50 mM HEPES/NaOH pH 7.5, 10 mM MgCl_2_, 0.05% Triton X-100, and 0.083 µg/µL cellosyl in a final volume of 50 µL. In high salt conditions, NaCl was added at 500 mM NaCl. In low salt conditions, no additional NaCl was added, and final concentration of NaCl depended on the amount carried by the protein stocks. Final NaCl concentration at low salt was 30 mM for PBP1B reactions and 45 mM for PBP1A reactions. When indicated, LpoA(sol) or LpoB(sol) were added at a concentration of 2 µM, and moenomycin was added at a concentration of 50 µM. Reactions were started by the addition of dansylated lipid II to a final concentration of 10 µM and monitored by following the decrease in fluorescence over 60 min at 25°C for PBP1B and over 90 min at 30°C for PBP1A, using a Clariostar plate reader (BMG Labtech, Germany) with excitation at 330 nm and emission at 520 nm.

### *In vitro* peptidoglycan synthesis assay using radiolabeled lipid II

To assay the *in vitro* PG synthesis activity of PBP1A and PBP1B with radiolabeled lipid II substrate, we used a previously published assay (14, 47). Final reactions included 50 mM HEPES/NaOH pH 7.5, 10 mM MgCl_2_, and 0.05% Triton X-100. As for the dansylated-lipid II GTase assay, NaCl was 500 mM in high salt conditions for both proteins and 30 and 45 mM for PBP1B and PBP1A reactions, respectively, in low salt conditions. The concentration of class A PBPs was 0.5 µM, and when indicated, LpoA(sol) and LpoB(sol) were added at 2 µM. Reactions were carried out for 1 h at 37°C. Reactions were stopped by boiling for 5 min. Digestion with cellosyl, reduction with sodium borohydride, and analysis by HPLC were performed as described (47).

### FRET-based *in vitro* peptidoglycan synthesis assay in detergents

Reactions were prepared in the same buffer conditions as the dansylated-lipid II GTase assay, with the exception of cellosyl which was absent in the assays. Samples contained 50 mM HEPES/NaOH pH 7.5, 10 mM MgCl_2_, and 0.05% Triton X-100 in a final volume of 50 µL, and NaCl concentration was 500 mM in high salt conditions for both PBP1B and PBP1A, and 30 and 45 mM for PBP1B and PBP1A reactions, respectively, in low salt conditions. PBP1B and PBP1A were added at a concentration of 0.5 µM. When indicated, activators LpoB(sol) or LpoA(sol) were added at a concentration of 2 µM. Reactions were started by the addition of an equimolar mix of lipid II, lipid II-Atto550 and lipid II-Atto647n, each at 5 µM and monitored by measuring fluorescence using a Clariostar plate reader (BMG Labtech, Germany) with excitation at 540 nm and emission measurements at 590 and 680 nm. Reactions were incubated at the indicated temperature for 60 or 90 min. After the reaction, emission spectra from 550 to 740 nm were taken in the same plate reader with excitation at 522 nm. When indicated, ampicillin was added at 1 mM and moenomycin was added at 50 µM. After plate reader measurements, reactions were stopped by boiling for 5 min, vacuum-dried using a speed-vac desiccator, and analyzed by Tris-Tricine SDS-PAGE as previously described (67).

## Data Availability

The raw experimental data of the chemical genomics screens and enzyme activity assays are available upon request from the corresponding authors. The ChemGAPP Small package used to support the chemical genetics findings of this study are openly available in GitHub. ChemGAPP is a Package for Chemical Genomic Analysis and Phenotypic Profiling.

## SUPPLEMENTAL MATERIAL

Supplemental material mbio.00325-24-s0001.pdf Supplemental figures and tables.

ASM does not own the copyrights to Supplemental Material that may be linked to, or accessed through, an article. The authors have granted ASM a non-exclusive, world-wide license to publish the Supplemental Material files. Please contact the corresponding author directly for reuse.
