# Supplementary material for: Reduced peptidoglycan synthesis capacity impairs growth of E. coli at high salt concentration
Source: mBio. 2024 Mar 1;15(4):e00325-24. doi: 10.1128/mbio.00325-24 (PMC11005333; doi:10.1128/mbio.00325-24)
Supplement: Supplemental material — Supplemental figures and tables. [file mbio.00325-24-s0001.pdf]

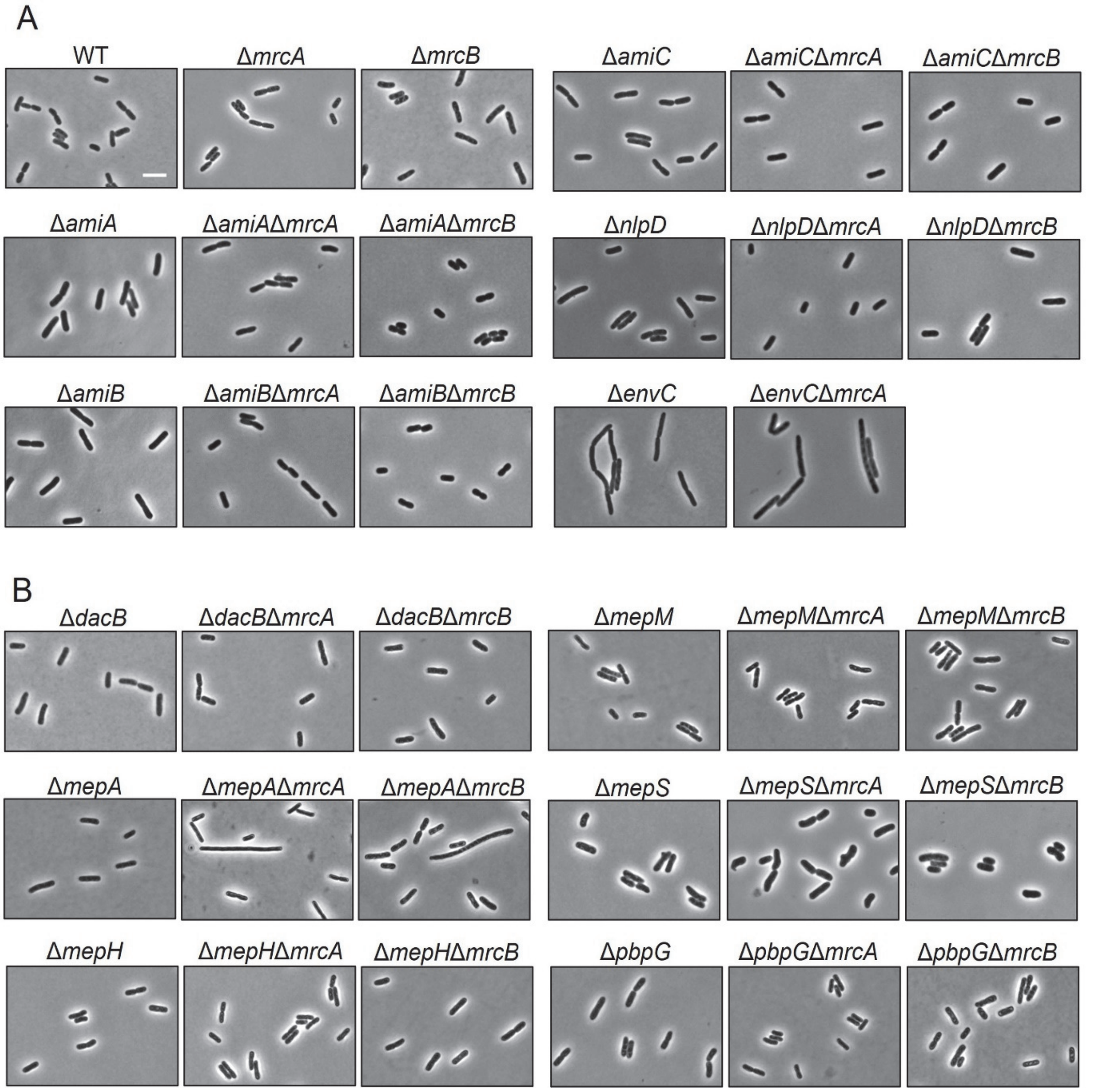

Figure S1. **Absence of the EPases PBP4 (encoded by *dacB*), MepM, MepA, MepS, MepH and PBP7 (encoded by *pbpG*) and amidases/regulators AmiA, AmiB, AmiC, NlpD and EnvC in Δ*mrcA* and Δ*mrcB* cells causes mild changes in cell morphology.** Phase contrast microscopy of **(A)** Morphology of *E. coli* BW25113 WT, Δ*mrcA,* Δ*mrcB,* Δ*amiA,* Δ*amiB,* Δ*amiC,* Δ*nlpD,* Δ*envC* double mutants lacking either *mrcA* or *mrcB.* **(B)** Morphology of Δ*dacB,* Δ*mepA,* Δ*mepH,* Δ*mepM,* Δ*mepS,* Δ*pbpG* mutants lacking either *mrcA* and *mrcB* in mid-exponential phase growth (OD_{600}=0.4) in LB medium at 37°C. Images of fluorescence microscopy are shown in Fig. S2. Scale Bar = 5 µm. Scale bar shown is representative for all images.

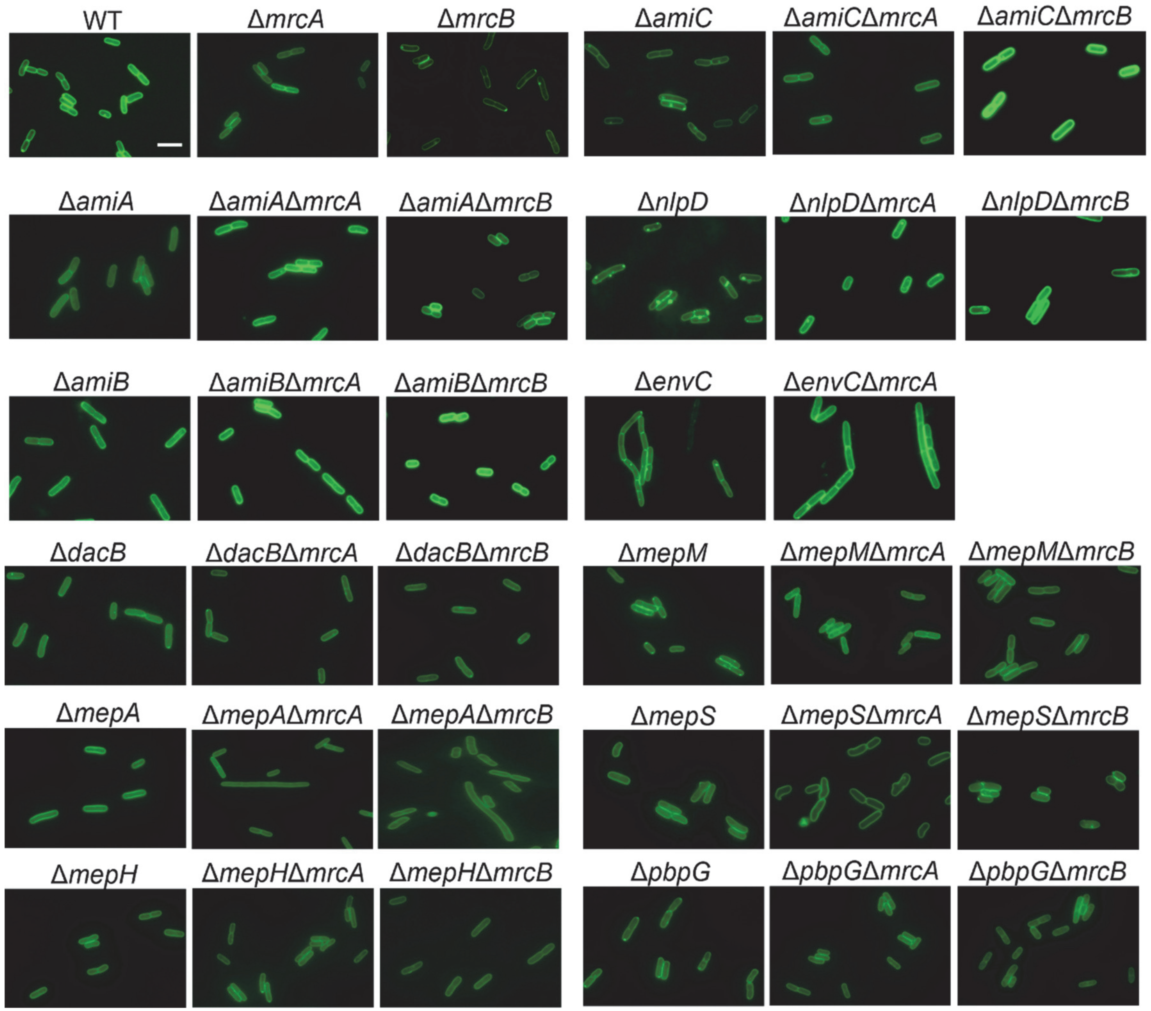

Figure S2a. Fluorescence microscopy of the WT, Δ*mrcA*, Δ*mrcB*, Δ*amiA*, Δ*amiB*, Δ*amiC*, Δ*nlpD*, Δ*envC*, Δ*dacB*, Δ*mepA*, Δ*mepH*, Δ*mepM*, Δ*mepS*, Δ*pbpG* and respective Δ*mrcA* and Δ*mrcB* double mutants. The cell cultures were grown in LB at 37°C until mid-exponential phase, stained with the membrane dye (A) FM1-43-FX, fixed, and visualised by fluorescence microscopy. Scale bar = 5 μm.

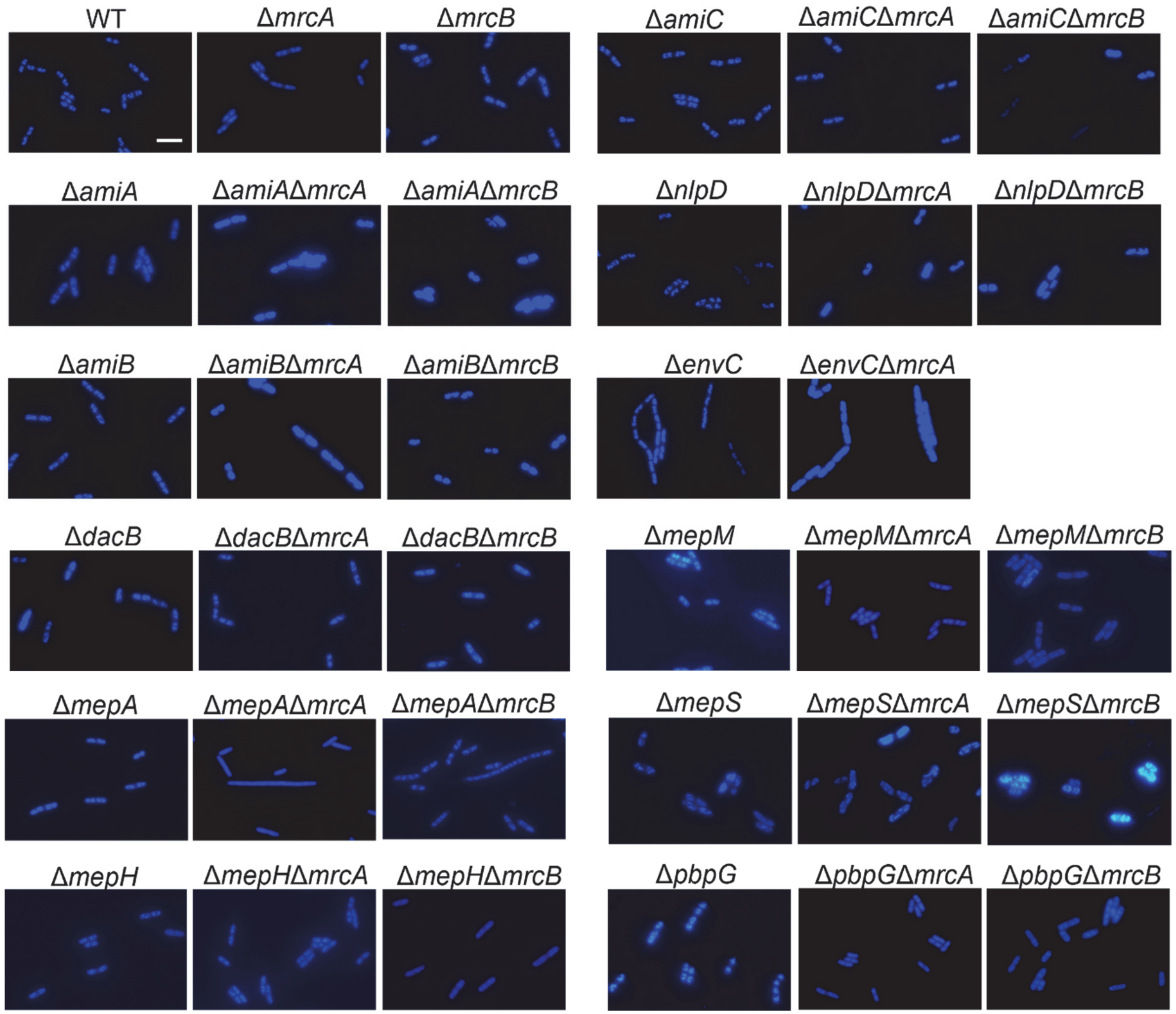

Figure S2b. Fluorescence microscopy of the WT, Δ*mrcA*, Δ*mrcB*, Δ*amiA*, Δ*amiB*, Δ*amiC*, Δ*nlpD*, Δ*envC*, Δ*dacB*, Δ*mepA*, Δ*mepH*, Δ*mepM*, Δ*mepS*, Δ*pbpG* and respective Δ*mrcA* and Δ*mrcB* double mutants. The cell cultures were grown in LB at 37°C until mid-exponential phase, stained with the membrane dye (A) FM1-43-FX, fixed, and visualised by fluorescence microscopy. Scale bar = 5 μm.

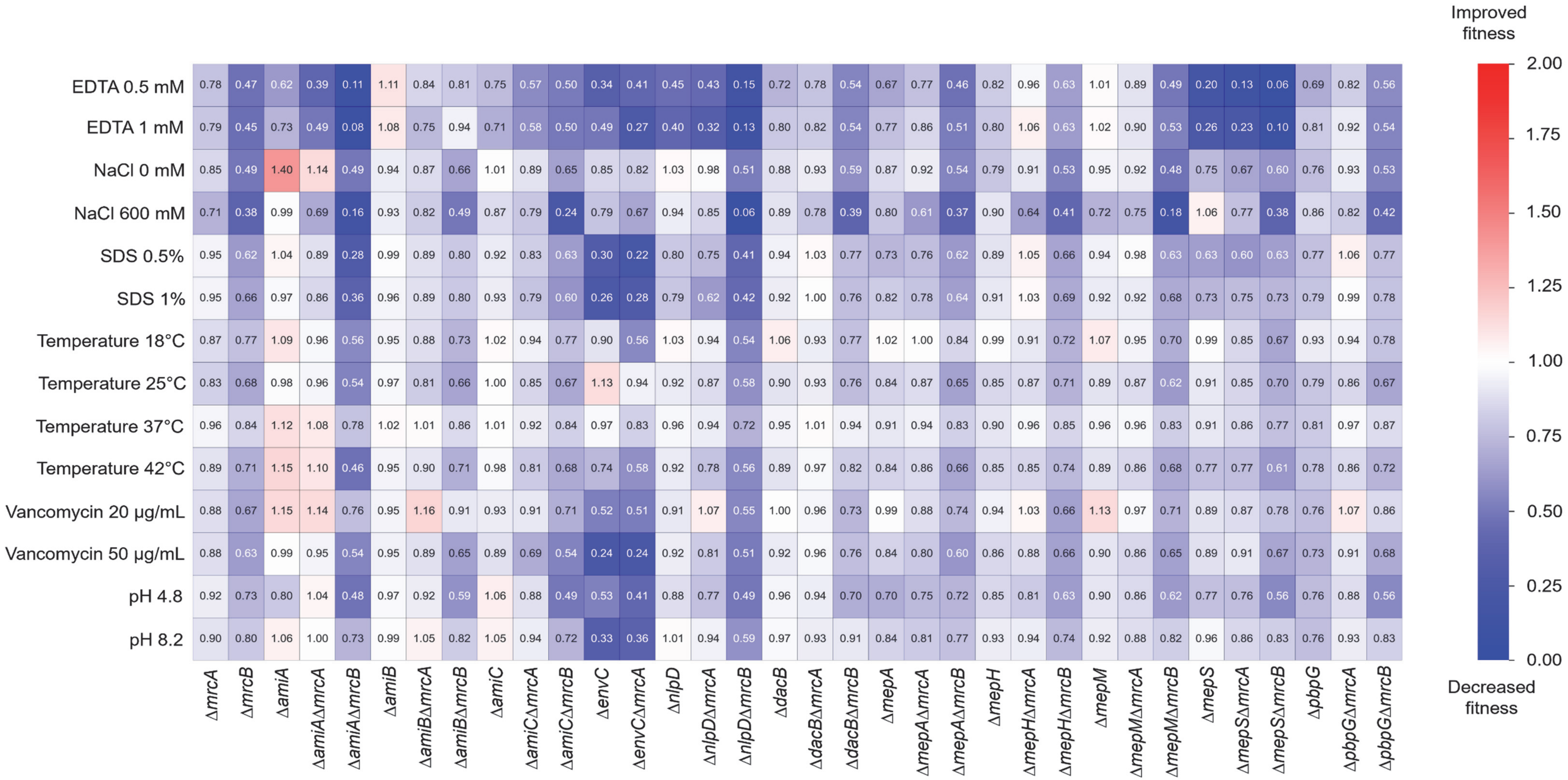

Figure S3. Chemical genetics screening indicates high and no NaCl conditions as the harshest stress for the mutants tested, causing decreased fitness. Heatmap of chemical genetics screening generated by ChemGAPP of Δ*mrcA*, Δ*mrcB*, EPase and amidase mutants under different conditions. Colony size was considered as fitness readout. The different colours represent the colony size compared to WT for each condition ranging from blue (smaller colony size and decreased fitness) through white (WT size) to red (larger colony size and increased fitness).

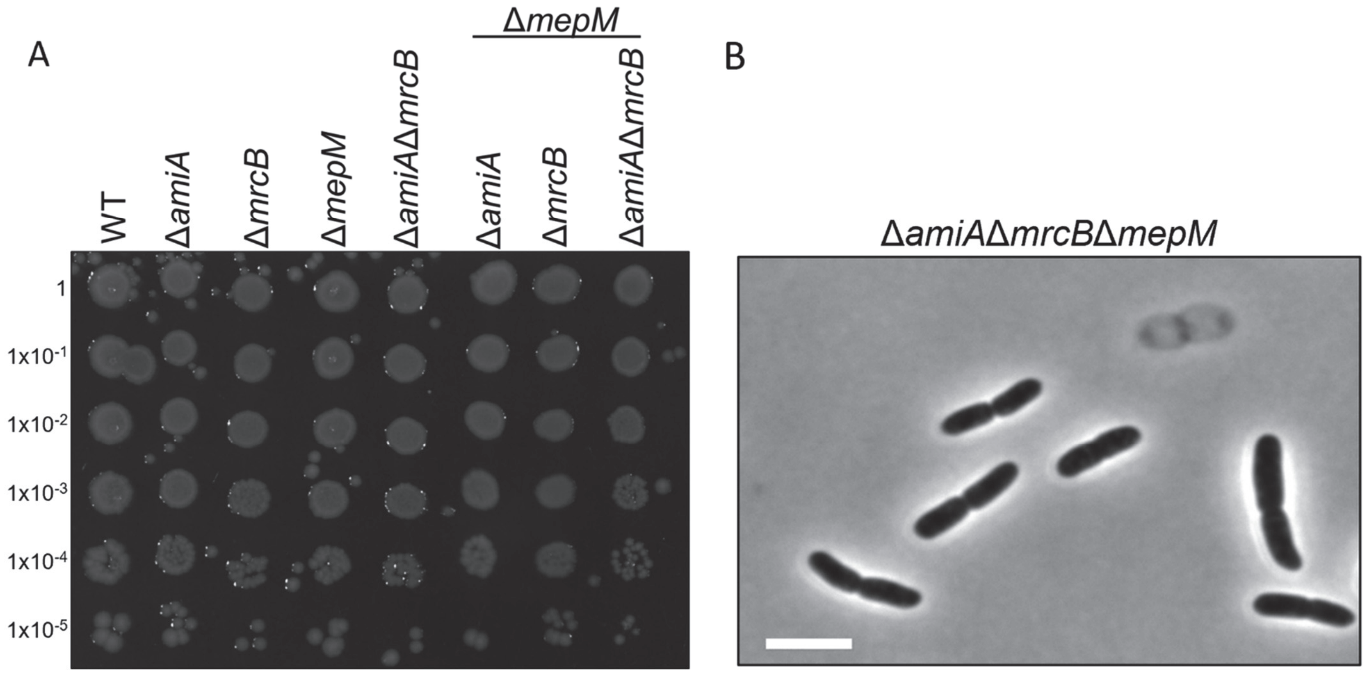

Figure S4. The triple mutant Δ*amiA*Δ*mrcB*Δ*mepM* is viable and shows a low number of lysed cells. (**A**) WT, Δ*amiA*, Δ*mrcB*, Δ*mepM*, Δ*amiA*Δ*mrcB*, Δ*amiA*Δ*mepM,* Δ*mrcB*Δ*mepM* and Δ*amiA*Δ*mrcB*Δ*mepM* strains were grown overnight in LB at 37°C. The OD600 of the cultures were adjusted to 1 and cultures were serially diluted (10^{-1} to 10^{-6}). Five µL of each dilution was spotted on LB agar. The plates were incubated at 37°C and photographed after ~16 h. (**B**) Phase contrast microscopy of Δ*amiA*Δ*mrcB*Δ*mepM* cells grown in LB at 37°C until mid-exponential phase (OD600=0.4). Scale bar = 5µm.

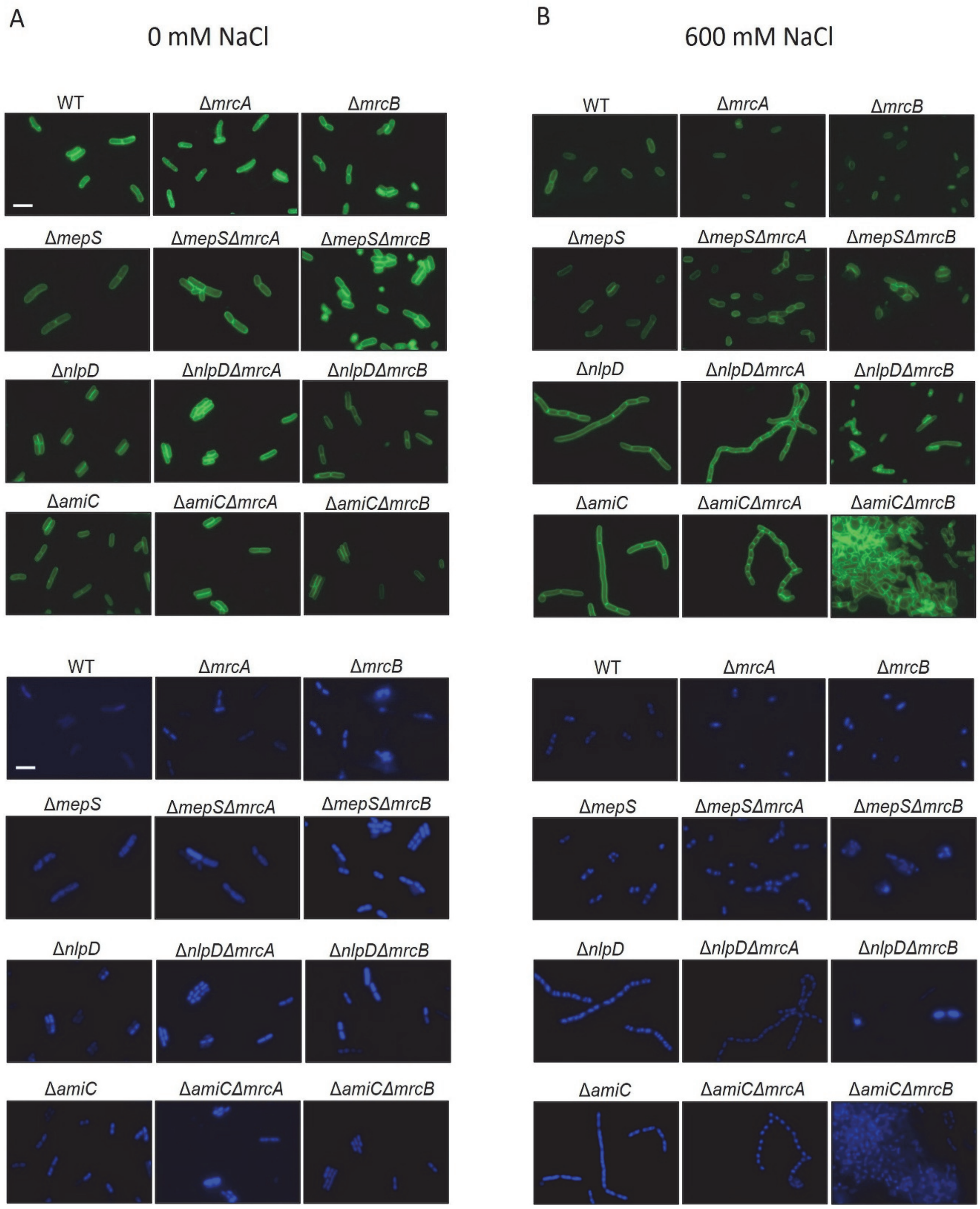

Figure S5. Fluorescence microscopy of WT, Δ*mrcA*, Δ*mrcB*, Δ*mepS*, Δ*nlpD*, Δ*amiC* and double mutant cells lacking either *mrcA* or *mrcB*. The cells were grown at 37°C in LB medium to early exponential phase (OD_{600}=~0.2), cultures were back-diluted into media with (**A**) 0 mM NaCl or (**B**) 600 mM NaCl and cells were grown until cultures reached an OD_{600} of ~0.2. Cells were stained with the membrane dye FM1-43-FX, fixed and stained with DAPI. The cells were visualised by fluorescence microscopy. Scale bar = 5 µm. Scale bar shown is representative for all images.

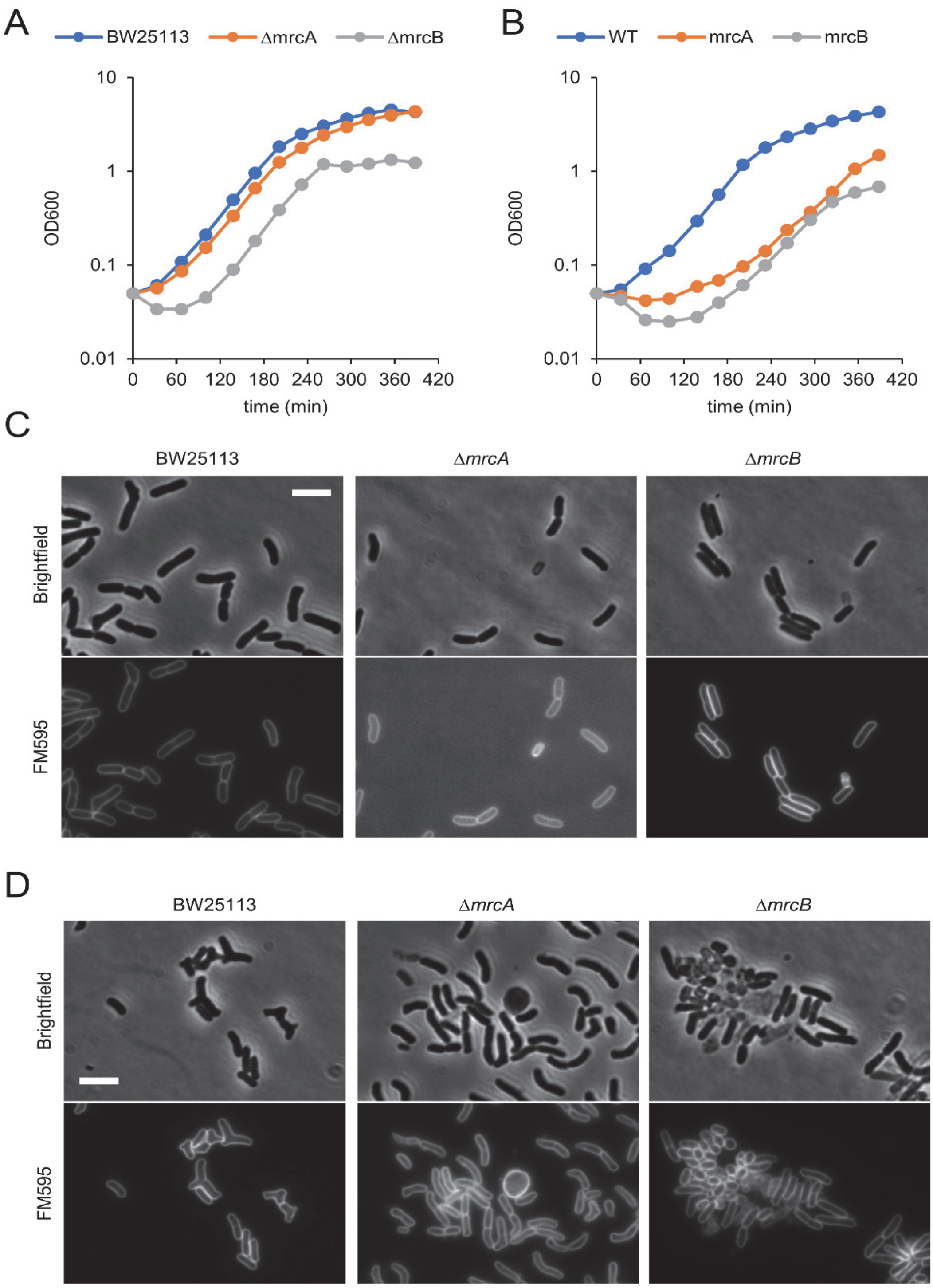

Figure S6. Effect of high salt on the growth of BW25113, Δ*mrcA* and Δ*mrcB*. Representative growth curves for BW25113 (WT), BW25113Δ*mrcA*::kan (Δ*mrcA*) and BW25113Δ*mrcB*::kan (Δ*mrcB*) grown in LB with 350 mM (**A**) or 500 mM (**B**) NaCl. (**C**) Representative microscopy images of strains grown in LB with 350 mM NaCl. Samples were taken at OD_{600} of 0.50 (WT), 0.33 (Δ*mrcA*) and 0.73 Δ*mrcB*. (**D**) Representative microscopy images of strains grown in LB with 500 mM NaCl. Samples were taken at OD_{600} of 1.80, WT, 0.37, Δ*mrcA*, and 0.30, Δ*mrcB*. Scale bar, 5 µm. Scale bar shown is representative for all images.

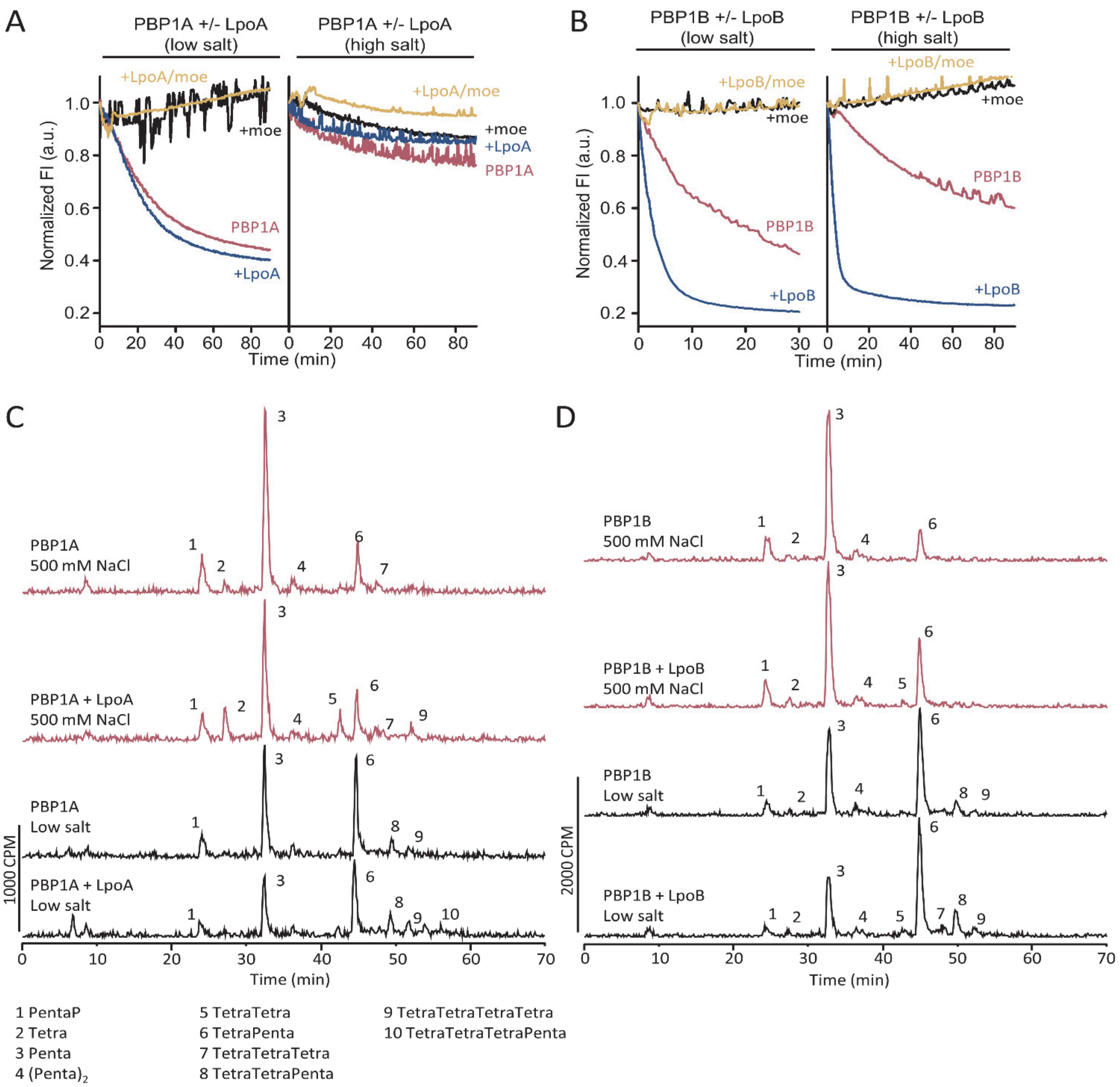

Figure S7. Activities of PBP1A and PBP1B is reduced at high salt concentrations. (**A**) Representative curves from real-time GTase assays with PBP1A at low and high salt conditions. Samples contained 0.5 µM PBP1A and 10 µM dansyl-lipid II, in the presence (blue, yellow) or absence (red, black) of 2 µM LpoA. When indicated, ampicillin (1 mM, black line) or moenomycin (50 µM, yellow line) were added. Samples were incubated for 90 min at 30°C. (**B**) Representative curves from GTase assays with PBP1B at low and high salt conditions. Samples contained 0.5 µM PBP1B in the presence (blue, yellow) or absence (red, black) of 2 µM LpoB. Same components and concentrations were added as in (**A**). Samples were incubated at 25°C for 30 min at low salt, and 90 min at high salt and contained 500 mM NaCl at high salt conditions, or 30 (45 mM) at low salt conditions for reactions with PBP1B (PBP1A). (**C**) HPLC analysis of the products of PG synthesis reactions with PBP1A (0.7 µM) and [^{14}C]-lipid II (25 µM). Reactions were carried out at two different salt concentrations, 500 mM NaCl (high salt, red) and 45 mM NaCl (low salt, black), and in the presence or absence of LpoA (2.8

μM), for 1 h at 37°C. (**D**) HPLC analysis of the products of PG synthesis reactions with PBP1B (0.5 μM) and [^{14}C]-lipid II (25 μM). Reactions were carried out at two different salt concentrations, 500 mM NaCl (high salt, red) and 30 mM NaCl (low salt, black), and in the presence or absence of LpoB (2 μM), for 1 h at 37°C. The identity of the muropeptide peaks is listed in (**C**).

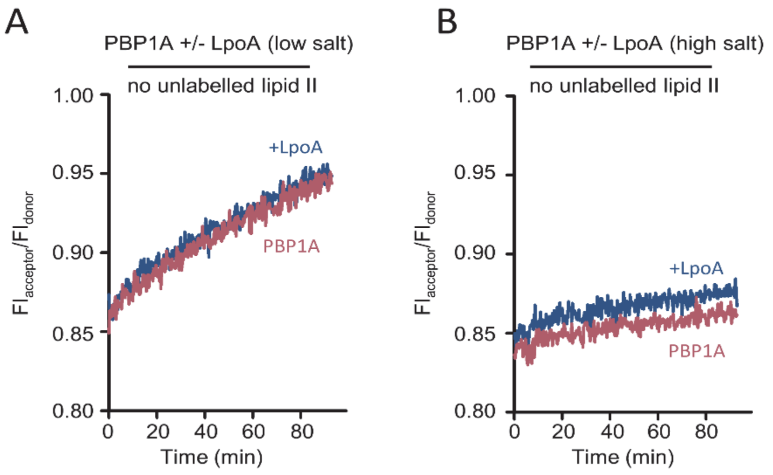

Figure S8. PBP1A is not able to efficiently incorporate Lipid II-Atto647n and Lipid II-Atto550 into long glycan chains in the absence of unlabelled lipid. Representative reaction curves from FRET assays of PBP1A at low (**A**) and high (**B**) salt conditions, respectively, performed in the absence of unlabelled lipid II. Samples contained 0.5 µM PBP1A, Atto550-labelled lipid II (5 µM) and Atto647n-labelled lipid II (5 µM), and LpoA (2 µM, blue line). Red lines indicate samples without LpoA. The conditions are the same as in Fig. 4C.

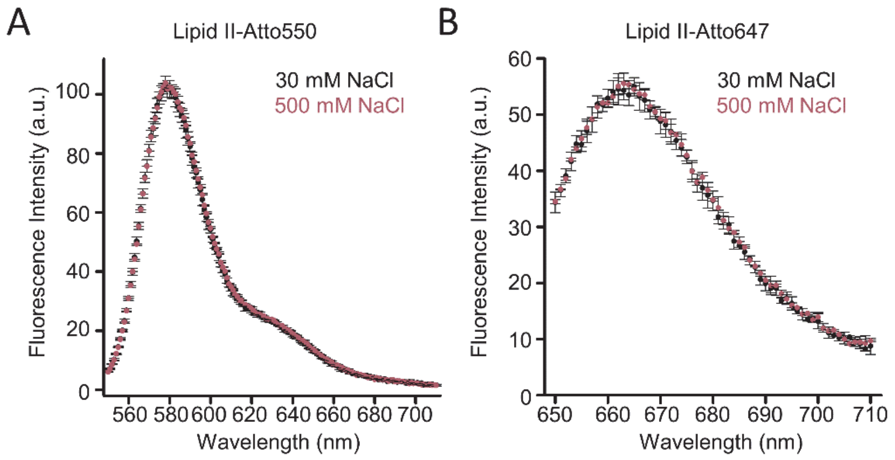

Figure S9. The fluorescence intensity of FRET assay labelled substrates does not change at high salt concentrations. Average of three fluorescence emission spectra of Atto550-labelled lipid II (A) or Atto647n-labelled lipid II (B) take at 30 or 500 mM NaCl. Both ligands were present at 5 µM and the buffer contained the same components as in activity assays at low or salt concentration. In A, spectra were taken with excitation at 526 nm and the emission was recorded from 550 to 710 nm. In B, spectra were taken with excitation at 622 nm and the emission was recorded from 650 to 710 nm.

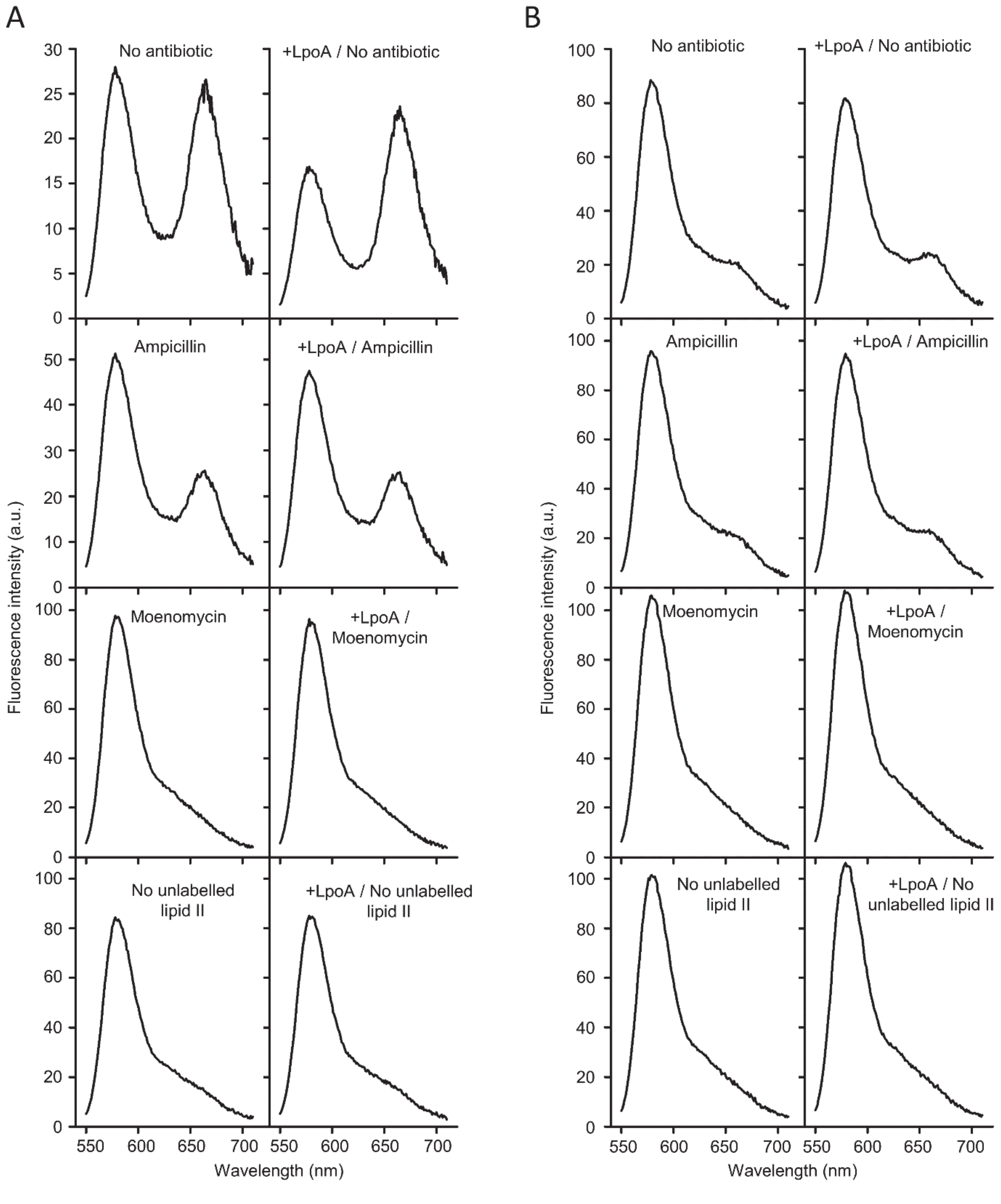

Figure S10. The PG synthesis activity of PBP1A is reduced at high salt concentrations. Fluorescence emission spectra taken at the end of reactions shown in Fig. 4C and Fig. S12 of reactions with low (**A**) or high (**B**) salt. Spectra were taken with excitation at 526 nm and the emission was recorded from 550 to 710 nm.

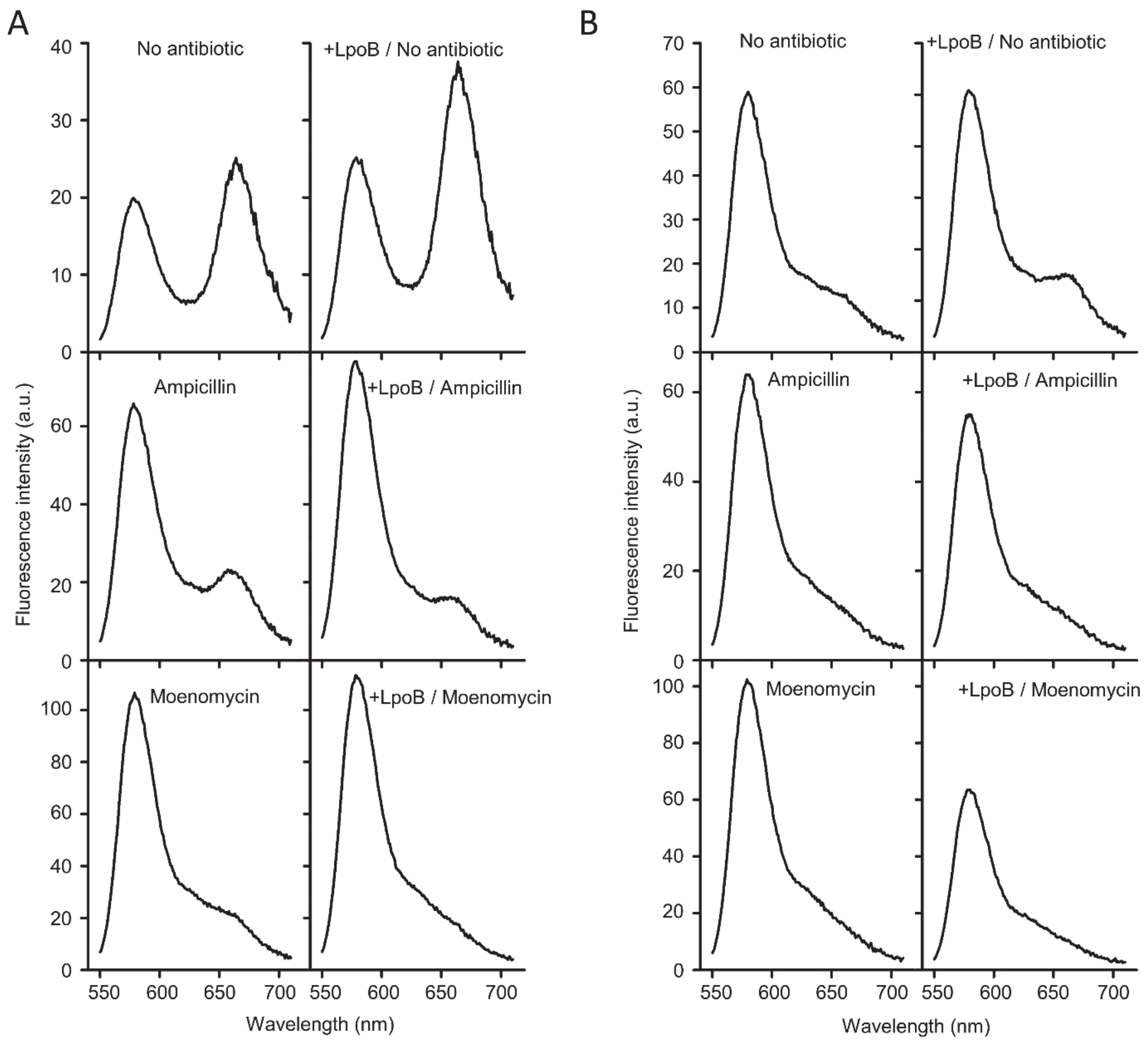

Figure S11. The PG synthesis activity of PBP1B is reduced at high salt concentrations. Fluorescence emission spectra taken at the end of reactions shown in Fig. 4C and Fig. S12 of reactions with low (**A**) or high (**B**) salt. Spectra were taken with excitation at 526 nm and the emission was recorded from 550 to 710 nm.

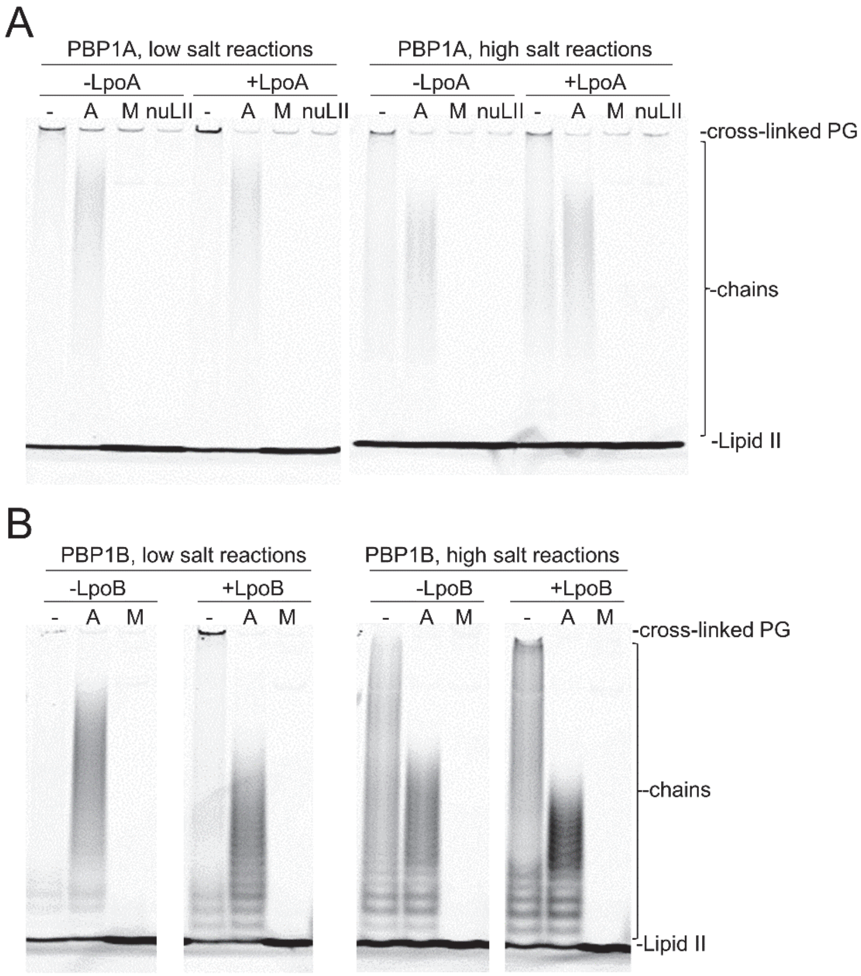

Figure S12. PG synthesis activity of PBP1A and PBP1B is reduced at high salt concentrations. (**A**) SDS-PAGE analysis of representative FRET assay curves from reactions with PBP1A at low and high salt conditions, in the presence or absence of LpoA. Conditions are the same as in Fig. 4C. (**B**) SDS-PAGE analysis of representative FRET assay samples of reactions with PBP1B at low and high salt conditions, in the presence or absence of LpoB. Conditions are the same as in Fig. 4D. In (**A**) and (**B**), lanes marked as '-' contain samples without an antibiotic; lanes labelled 'A' contain samples with ampicillin and lanes labelled 'M' contain samples with moenomycin. 'nuLII' indicates that samples did not contain unlabelled lipid II.

Table S1. Bacterial strains used in this study.

Strain ID	Genotype	Source
MB01064	BW25113	(67)
MB01119	BW25113 Δ*amiA::kan*	(34)
MB01120	BW25113 Δ*amiB::kan*	(34)
MB01121	BW25113 Δ*amiC::kan*	(34)
MB01122	BW25113 Δ*envC::kan*	(34)
MB01051	BW25113 Δ*nlpD::kan*	(34)
MB01118	BW25113 Δ*amiA::kan*Δ*mrcA::tet*	This study
MB01119	BW25113 Δ*amiB::kan*Δ*mrcA::tet*	This study
MB01120	BW25113 Δ*amiC::kan*Δ*mrcA::tet*	This study
MB01121	BW25113 Δ*envC::kan*Δ*mrcA::tet*	This study
MB01122	BW25113 Δ*nlpD::kan*Δ*mrcA::tet*	This study
MB01123	BW25113 Δ*amiA::kan*Δ*mrcB::tet*	This study
MB01124	BW25113 Δ*amiB::kan*Δ*mrcB::tet*	This study
MB01125	BW25113 Δ*amiC::kan*Δ*mrcB::tet*	This study
MB01126	BW25113 Δ*nlpD::kan*Δ*mrcB::tet*	This study
MB10098	BW25113 *ΔpbpG*	(22)
MB10502	BW25113 *ΔmepS::kan*	(22)
MB10075	BW25113 *ΔmepM::kan*	(22)
MB10397	BW25113 *ΔmrcA::tet*	(22)
MB10398	BW25113 *ΔmrcB::tet*	(22)
MB10067	BW25113 *ΔmepA::kan*	(22)
MB10023	BW25113 *ΔdacB::kan*	(22)
MB10020	BW25113 Δ*mepH*::kan	This study
MB10718	BW25113 *ΔmepS::kanΔmrcA::tet*	This study
MB10714	BW25113 *ΔmepA::kanΔmrcA::tet*	This study
MB10716	BW25113 *ΔmepH::kanΔmrcA::tet*	This study
MB10715	BW25113 Δ*pbpG::catΔmrcA::tet*	This study
MB10719	BW25113 Δ*mepM::kanΔmrcA::tet*	This study
MB10717	BW25113 Δ*dacB::kanΔmrcA::tet*	This study
MB10724	BW25113 Δ*mepS::kanΔmrcB::tet*	This study
MB10720	BW25113 Δ*mepA::kanΔmrcB::tet*	This study
MB10722	BW25113 Δ*mepH::kanΔmrcB::tet*	This study
MB10721	BW25113 Δ*pbpG::catΔmrcB::tet*	This study
MB10725	BW25113 Δ*mepM::kanΔmrcB::tet*	This study
MB10723	BW25113 Δ*dacB::kanΔmrcB::tet*	This study

Table S2. Phenotypes of *mrcA, mrcB*, amidases and EPases mutants in LB.

Relevant genotype	No. of cells[1]	Total length (µm)[2]	Avg length (µm)[3]	Total width (µm)[4]	Avg width (µm)[5]	Total no. of septa[6]	Length/ septum (µm)[7]	Length/ Segment (µm)[8]	No. of septa/cell[9]	No. of chaining cells[10]	% of chaining cells[11]	*P*-value avg. length[12]	*P*-value avg. width[13]	*P*-value no. of septa/cell[14]
WT	445	1594.2	3.6 ± 0.9	441.0	0.99 ± 0.1	179	8.9	2.6	0.4	0	0			
Δ*mrcA*	249	931.3	3.7 ± 1.0	224.9	0.90 ± 0.2	127	7.3	2.5	0.5	3	1.2	<0.05	<0.05	<0.05
Δ*mrcB*	260	982.5	3.8 ± 1.0	240.6	0.93 ± 0.1	128	7.7	2.5	0.5	5	1.9	<0.05	<0.05	<0.05
Δ*amiA*	177	947.5	5.4 ± 1.3	187.6	1.06 ± 0.1	100	9.5	3.4	0.6	4	2.3	<0.05	<0.05	<0.05
Δ*amiA*Δ*mrcA*	204	769.0	3.8 ± 1.1	175.9	0.86 ± 0.1	99	7.8	2.5	0.5	3	1.5	<0.05	<0.05	N.S
Δ*amiA*Δ*mrcB*	226	600.6	2.7 ± 0.9	215.6	0.95 ± 0.1	78	7.7	2.0	0.3	7	3.1	<0.05	<0.05	NS
Δ*amiB*	167	824.6	4.9 ± 1.0	176.1	1.05 ± 0.1	82.0	10.1	3.3	0.5	0	0	<0.05	<0.05	NS
Δ*amiB*Δ*mrcA*	180	662.5	3.7 ± 1.4	158.9	0.88 ± 0.1	108	6.1	2.3	0.6	8	4.4	NS	<0.05	<0.05
Δ*amiB*Δ*mrcB*	245	629.6	2.6 ± 0.8	228.6	0.93 ± 0.1	74	8.5	2.0	0.3	5	2.0	<0.05	<0.05	<0.05
Δ*amiC*	286	1441.7	5.0 ± 1.4	296.9	1.04 ± 0.1	142	10.2	3.4	0.5	0	0	<0.05	<0.05	<0.05
Δ*amiC*Δ*mrcA*	259	970.6	3.7 ± 2.1	240.5	0.93 ± 0.1	150	6.5	2.4	0.6	10	3.9	NS	<0.05	<0.05
Δ*amiC*Δ*mrcB*	137	515.3	3.8 ± 1.1	130.7	0.95 ± 0.1	73	7.1	2.5	0.5	2	1.5	NS	<0.05	<0.05
Δ*envC*	294	2111.0	7.2 ± 4.5	282.3	0.96 ± 0.1	481	4.4	2.7	1.6	110	37.4	<0.05	<0.05	<0.05
Δ*envC*Δ*mrcA*	143	947.3	6.6 ± 5.4	116.7	0.82 ± 0.1	144	6.6	3.3	1.0	26	18.2	<0.05	<0.05	<0.05
Δ*envC*Δ*mrcB*	ND	ND	ND	ND	ND	ND	ND	ND	ND	ND	ND	ND	ND	ND
Δ*nlpD*	218	1018.5	4.7 ± 1.2	225.7	1.04 ± 0.1	89	3.3	1.9	0.4	0	0	<0.05	<0.05	NS
Δ*nlpD*Δ*mrcA*	180	619.1	3.4 ± 1.3	161.9	0.90 ± 0.1	61	10.1	2.6	0.3	1	0.6	NS	<0.05	NS
Δ*nlpD*Δ*mrcB*	136	496.3	3.6 ± 0.9	133.4	0.98 ± 0.1	53	9.4	2.6	0.4	1	0.7	NS	NS	NS
Δ*dacB*	338	1413.7	4.2 ± 1.1	354.6	1.05 ± 0.1	146	9.7	2.9	0.4	4	1.2	<0.05	<0.05	NS
Δ*dacB*Δ*mrcA*	431	1770.7	4.1 ± 1.0	420.9	0.98 ± 0.1	238	7.4	2.6	0.6	6	1.4	<0.05	<0.05	<0.05
Δ*dacB*Δ*mrcB*	261	1083.8	4.2 ± 1.4	274.7	1.05 ± 0.1	123	8.8	2.8	0.5	8	3.1	<0.05	<0.05	NS
Δ*mepA*	243	1062.4	4.4 ± 1.7	231.2	0.95 ± 0.1	170	6.6	2.7	0.7	14	5.8	<0.05	<0.05	<0.05
Δ*mepA*Δ*mrcA*	407	1999.3	4.9 ± 3.4	315.3	0.77 ± 0.1	286	7.0	2.9	0.7	42	10.3	<0.05	<0.05	<0.05
Δ*mepA*Δ*mrcB*	483	2929.3	6.1 ± 4.4	447.8	0.93 ± 0.1	137	21.4	4.7	0.3	62	12.8	<0.05	<0.05	<0.05
Δ*mepH*	322	1253.0	3.9 ± 1.1	291.2	0.90 ± 0.1	175	7.2	2.5	0.5	2	0.6	<0.05	<0.05	<0.05
Δ*mepH*Δ*mrcA*	428	1529.7	3.6 ± 1.0	344.2	0.80 ± 0.1	221	6.9	2.4	0.5	5	1.2	NS	<0.05	<0.05
Δ*mepH*Δ*mrcB*	509	1945.8	3.8 ± 1.0	481.3	0.95 ± 0.1	207	9.4	2.7	0.4	0	0.0	<0.05	<0.05	NS
Δ*mepM*	332	1222.4	3.7 ± 0.9	312.1	0.94 ± 0.1	169	7.2	2.4	0.5	2	0.6	NS	<0.05	<0.05
Δ*mepM*Δ*mrcA*	467	1724.0	3.7 ± 0.9	399.3	0.85 ± 0.1	224	7.7	2.5	0.5	2	0.4	NS	<0.05	<0.05
Δ*mepM*Δ*mrcB*	330	1312.3	4.0 ± 1.1	320.5	0.97 ± 0.1	157	8.4	2.7	0.5	1	0.3	<0.05	<0.05	<0.05

Δ*mepS*	232	1053.6	4.5 ± 1.4	260.3	1.12 ± 0.1	115	9.2	3.0	0.5	3	1.3	<0.05	<0.05	<0.05
Δ*mepS*Δ*mrcA*	604	2860.8	4.7 ± 2.1	727.0	1.20 ± 0.1	245	11.7	3.4	0.4	23	3.8	<0.05	<0.05	NS
Δ*mepS*Δ*mrcB*	231	952.4	4.1 ± 1.4	276.0	1.19 ± 0.2	89	10.7	3.0	0.4	3	1.3	<0.05	<0.05	NS
Δ*pbpG*	225	885.4	3.9 ± 1.0	248.5	1.10 ± 0.1	90	9.8	2.8	0.4	0	0.0	<0.05	<0.05	<0.05
Δ*pbpG*Δ*mrcA*	260	864.7	3.3 ± 0.8	229.0	0.88 ± 0.1	101	8.6	2.4	0.4	2	0.8	<0.05	<0.05	NS
Δ*pbpG*Δ*mrcB*	203	802.7	4.0 ± 1.0	198.9	0.98 ± 0.1	108	7.4	2.6	0.5	1	0.5	<0.05	NS	<0.05

1. All cells were considered single cells independent of the number of segments.
2. Total length means cumulative length of all cells measured.
3. Refers to the total length/number of cells.
4. Total width means cumulative width of all cells measured.
5. Refers to the total width/number of cells.
6. Septa are considered as any membrane constrictions or completed membrane septa in cell chains.
7. Length/septum indicates the total length/total number of septa. It shows the frequency at which septa are detected. The number is much lower in chaining cells because septa persist for an abnormally long time.
8. The number of cell segments refers to the number of cells plus the number of septa. The “length/segment” is the total length/total number of segments. In normal (nonchaining) cells, this measurement is similar to the average cell length (pole-pole distance), but the value is smaller because predivisional cells contain two segments and are counted as two cells instead of one (i.e., some pole-to-septa measurements are taken into account, as well as pole-pole measurements). In chaining cells, the length/segment measurement refers mainly to the distance between adjacent septa.
9. Refers to total number of septa/number of cells.
10. Cells with more than one septum are considered chaining cells.
11. Percentage of chaining cells related to the total no. of cells.
12. *P*-values of Student t-test comparing the average length of WT cells with every mutant.
13. *P*-values of Student t-test test comparing the average width of WT cells with every mutant.
14. *P*-values of Student t-test test comparing number of septa/cell of WT cells with every mutant.

NS, not significant (significance indicated by Student's t- test *P* value)

ND, not detected

Table S3. Phenotypes of *mrcA*, *mrcB*, *mepS, amiC* and *nlpD* mutants in LB with 600 mM NaCl or 0 mM NaCl.

Relevant genotype	Total no. of cells[1]	No. of viable cells[2]	Total length (µm)[3]	Avg length (µm)[4]	Total width (µm)[5]	Avg width (µm)[6]	Total no. of septa[7]	No. of septa/cell[8]	No. of chaining cells[9]	% of chaining cells[10]	No. of lysed cells[11]	% of lysed cells[12]	*P*-value avg. length[13]	*P*-value avg. width[14]	*P*-value no. of septa/cell[15]
**600 mM NaCl**															
WT	315	315	1108.1	3.5 ± 1.3	335.9	1.07 ± 0.2	126	1.5	0	0	0	0			
Δ*mrcA*	357	357	803.2	2.2 ± 0.8	347.0	0.97 ± 0.3	62.0	2.2	0	0	0	0	<0.05	<0.05	<0.05
Δ*mrcB*	501	421	1180.2	2.8 ± 0.8	487.9	1.16 ± 0.2	178.0	ND	0	0	80	16	<0.05	<0.05	N.S
Δ*mepS*	348	348	1505.9	4.3 ± 2.0	371.1	1.07 ± 0.2	197	0.6	0	0	0	0	<0.05	N.S	<0.05
Δ*mepS*Δ*mrcA*	455	455	1625.6	3.6 ± 1.5	478.9	1.05 ± 0.2	302	0.7	0	0	0	0	N.S	N.S	<0.05
Δ*mepS*Δ*mrcB*	441	263	847.2	3.2 ± 1.1	274.9	1.23 ± 0.3	113	0.5	0	0	178	40	N.S	<0.05	N.S
Δ*amiC*	258	258	3152.8	12.2 ± 8.5	259.6	1.01 ± 0.1	538.0	2.1	127	49.2	0	0	<0.05	<0.05	<0.05
Δ*amiC*Δ*mrcA*	160	131	1306.7	10.0 ± 6.5	124.3	0.95 ± 0.3	439	3.4	126	96.2	29	18.1	<0.05	<0.05	<0.05
Δ*amiC*Δ*mrcB*	ND	ND	ND	ND	ND	ND	ND	ND	ND	ND	ND	ND	ND	ND	ND
Δ*nlpD*	165	165	2597.3	15.7 ± 12.1	162.5	0.99 ± 0.1	569	3.4	108	65.5	0	0	<0.05	<0.05	<0.05
Δ*nlpD*Δ*mrcA*	108	108	1748.7	16.2 ± 9.5	114.5	1.06 ± 0.7	614	5.7	103	95.4	0	0	<0.05	N.S	<0.05
Δ*nlpD*Δ*mrcB*	ND	ND	ND	ND	ND	ND	ND	ND	ND	ND	ND	ND	ND	ND	ND
**0 mM NaCl**															
WT	288	288	1212.8	4.2 ± 1.3	286.2	0.99 ± 0.2	152	0.53	0	0	0	0			
Δ*mrcA*	458	458	1800.8	3.9 ± 1.2	430.4	0.94 ± 0.2	219	0.48	0	0	0	0	<0.05	<0.05	NS
Δ*mrcB*	346	298	1128.2	3.8 ± 0.9	299.3	1.00 ± 0.1	154	0.52	0	0	48	16	<0.05	NS	NS
Δ*mepS*	214	214	1050.7	4.9 ± 1.6	285.8	1.34 ± 0.2	120	0.56	0	0	0	0	<0.05	<0.05	NS
Δ*mepS*Δ*mrcA*	304	304	1501.5	4.9 ± 2.0	380.9	1.25 ± 0.2	187	0.62	0	0	0	0	<0.05	<0.05	NS
Δ*mepS*Δ*mrcB*	499	499	1963.8	3.9 ± 1.3	581.0	1.16 ± 0.1	203	0.41	0	0	ND	ND	<0.05	<0.05	<0.05
Δ*amiC*	284	284	1103.8	3.9 ± 1.1	272.1	0.96 ± 0.1	131	0.5	0	0	0	0	<0.05	<0.05	NS
Δ*amiC*Δ*mrcA*	269	269	1005.3	3.7 ± 0.9	246.7	0.92 ± 0.1	139	0.5	0	0	0	0	<0.05	<0.05	NS
Δ*amiC*Δ*mrcB*	398	398	1578.6	4.0 ± 1.1	383.2	0.96 ± 0.1	251	0.6	27	6.8	0	0	<0.05	<0.05	NS
Δ*nlpD*	317	317	1253.8	4.0 ± 1.0	300.5	0.95 ± 0.1	173	0.5	0	0	0	0	<0.05	<0.05	NS
Δ*nlpD*Δ*mrcA*	223	223	849.0	3.8 ± 0.9	199.4	0.89 ± 0.1	110	0.5	5	2.2	0	0	<0.05	<0.05	NS
Δ*nlpD*Δ*mrcB*	429	389	1781.6	4.6 ± 1.9	379.8	0.98 ± 0.1	247	0.6	8	2.1	40	9.3	<0.05	NS	<0.05

1. Total number refers to the sum of viable cells and lysed cells.
2. Viable cells were considered non-lysed cells.
3. Total length means cumulative length of all cells measured.
4. Refers to the total length/number of cells.
5. Total width means cumulative width of all cells measured.
6. Refers to the total width/number of cells.
7. Septa are considered as any membrane constrictions or completed membrane septa in cell chains.

8. Refers to total number of septa/number of cells.
9. Cells with more than one septum are considered chaining cells.
10. Percentage of chaining cells related to the total no. of cells.
11. Phase light cells were considered as lysed cells.
12. Percentage of lysed cells related to the total number of cells.
13. *P*-values of Student t-test comparing the average length of WT cells with every mutant.
14. *P*-values of Student t-test test comparing the average width of WT cells with every mutant.
15. *P*-values of Student t-test test comparing number of septa/cell of WT cells with every mutant.

NS, not significant (significance indicated by Student's t test or Chi-square test *P* value)

ND, not detected. The number of lysed cells was too high for counting.
